# The impact of pectin supplementation on systemic inflammation pathways, gut microbiome, and metabolic health in patients with Metabolic Dysfunction-Associated Steatotic Liver Disease (MASLD): A study protocol for a randomised controlled trial

**DOI:** 10.1371/journal.pone.0352397

**Published:** 2026-07-13

**Authors:** Noor Kifah Al-Tameemi, Jane I. Grove, Caroline L. Hoad, Jessica Wong Sun Wai, Adina Olaru, Saikat Mandal, Katie E. Rollins, Christopher R. Bradley, Susan T. Francis, Penny A. Gowland, Ana M. Valdes, Guruprasad P. Aithal

**Affiliations:** 1 School of Medicine, University of Nottingham, Nottingham, United Kingdom; 2 National Institute of Health Research (NIHR) Nottingham Biomedical Research Centre, Nottingham University Hospitals NHS Trust and the University of Nottingham, Nottingham, United Kingdom; 3 Sir Peter Mansfield Imaging Centre, School of Physics and Astronomy, University of Nottingham, Nottingham, United Kingdom; Instituto Nacional de Ciencias Medicas y Nutricion Salvador Zubiran, MEXICO

## Abstract

**Background:**

Metabolic dysfunction–associated steatotic liver disease (MASLD) is the leading cause of chronic liver disease, affecting over 30% of adults worldwide. Emerging evidence suggests that dietary fibre, particularly pectin, may improve metabolic health by modulating inflammation, gut microbiota composition, and intestinal permeability. However, controlled human studies in MASLD are limited. This study aims to evaluate the effect of pectin supplementation on systemic inflammation, gut microbiome, and metabolic health in patients with MASLD.

**Methods:**

This single-centre, double-blind, randomised, placebo-controlled dietary intervention will be conducted at Nottingham University Hospitals NHS Trust in partnership with the University of Nottingham. Thirty adults with MASLD will be randomised (1:1) to receive either 15g/day Low-methoxyl (LM) pectin or a matched placebo for six weeks. Each participant will attend baseline and post-intervention visits during which anthropometric data, fasting blood samples, and stool samples will be collected. FibroScan® assessments will be performed for all participants at both visits to quantify liver stiffness and steatosis. Twenty-two participants will take part in a magnetic resonance imaging (MRI) sub-study to evaluate hepatic and intestinal characteristics at baseline and post-intervention. Laboratory analyses will include liver function, lipid, glycemic, and inflammatory markers, alongside profiling of gut microbiota composition and short-chain fatty acids.

**Discussion:**

This is the first randomised controlled study to evaluate the mechanistic effects of pectin supplementation on inflammation, gut microbiome composition, and metabolic outcomes in MASLD. The results may generate novel evidence on the role of soluble fibre in modulating the gut–liver axis and support the development of scalable, nutrition-based interventions to improve metabolic and hepatic health in this population.

**Clinical trial registration:**

The trial was registered on ClinicalTrials.gov (Identifier: NCT07093346)

## Introduction

### Background and rationale

Metabolic dysfunction–associated steatotic liver disease (MASLD) is now recognised as a major global health concern [1]. MASLD is defined as a spectrum of liver fat accumulation linked to metabolic dysfunction, rather than excessive alcohol intake. Currently, more than 30% of adults worldwide are affected by MASLD, and it is considered one of the leading causes of liver transplantation worldwide. The prevalence is even higher among individuals who are overweight, have insulin resistance, or have type 2 diabetes [2].

MASLD represents a continuum, ranging from simple fat accumulation in the liver to metabolic dysfunction–associated steatohepatitis (MASH), progressing to fibrosis, cirrhosis, and, in some cases, hepatocellular carcinoma or liver failure [2].

The pathogenesis of MASLD has been linked to the presence of one or more of the following factors: impaired lipid metabolism, insulin resistance, increased gut permeability, high-starch carbohydrates and high-fat diets, genetic factors, alterations in inflammatory signaling and immune system pathways, endoplasmic reticulum stress, and oxidative stress. All these factors play an important role in the progression of MASLD to more severe stages of liver disease [3,4].

Growing research suggests a link between gut health and the development of various liver conditions, such as MASLD and MASH. This is based on findings indicating that individuals with MASLD exhibit higher intestinal permeability, endotoxemia, and increased bacterial overgrowth in the small intestine compared to those without the condition. Additionally, liver disease has been linked to alterations in gut bacterial composition, and modification of the gut flora has been shown to influence liver damage [5,6].

Dietary modification remains the cornerstone of management [7]. However, long-term adherence remains suboptimal due to multiple barriers, including restrictive meal plans, low motivation, limited nutritional knowledge, and insufficient sustained support.

Dietary fibres refer to the portion of plant-derived carbohydrates and lignin that cannot be digested or hydrolysed by digestive enzymes in the human small intestine and may undergo partial or complete fermentation in the gut. While fibres are not directly absorbed or metabolised by human enzymes, they play a critical physiological role in gastrointestinal health, microbiome dynamics, metabolic regulation, and immune modulation [8,9].

Among soluble fibres, pectin has emerged as a particularly promising fibre for therapeutic applications [10]. Pectin is a complex soluble heteropolysaccharide that is found in the primary cell walls of fruits such as apples, citrus peels, and plums and is commonly used as a food additive. Pectin is classified based on the esterification degree to either high-methoxyl (HM) pectin, where the esterification degree is higher than 50% or Low-methoxyl (LM) pectin, where the esterification degree is lower than 50%. HM pectin is generally less readily fermentable by gut microbiota, whereas LM pectin (lower DE) is more easily fermented in the colon. This fermentability difference is primarily driven by structural accessibility to microbial enzymes. LM pectin fermentation results in the production short-chain fatty acids (SCFAs), notably acetate, propionate, and butyrate, which serve as energy substrates and signaling molecules influencing inflammation, lipid metabolism, and gut-barrier function. Moreover, pectin demonstrates broad-spectrum effects on microbial composition, gut barrier integrity, and systemic immune modulation [11,12].

LM pectin has been shown to reduce hepatic lipid accumulation, enhance gut barrier integrity, and attenuate inflammatory signalling in preclinical studies [13,14]. Moreover, a pilot clinical trial reported that supplementation with 15−20 g/day of LM pectin significantly reduced circulating pro-inflammatory cytokines, including tumour necrosis factor-alpha (TNF-α), interferon-gamma (IFN-γ), interleukin-1 beta (IL-1β) and interleukin 6, as well as increased the anti-inflammatory cytokine interleukin 10 (IL-10) in healthy adults. In parallel, significant improvements were noted in psychological wellbeing, with decreased anxiety and depression scores measured using the Hospital Anxiety and Depression Scale (HADS) (p < 0.05). No significant changes in gastrointestinal symptoms were reported, indicating good tolerability of LM pectin supplementation [15].

These immunomodulatory effects are thought to be mediated, at least in part, through microbial fermentation of LM pectin and subsequent production of short-chain fatty acids, which may regulate immune signalling pathways, including NF-κB activity, and support gut barrier integrity, thereby contributing to systemic anti-inflammatory effects [15].

Despite these promising findings, the effects of purified pectin supplementation on hepatic and metabolic outcomes in individuals with MASLD have not been rigorously tested via randomised controlled trials.

Therefore, the primary aim of this research is to determine the effects of daily LM pectin ingestion on inflammatory pathways that drive disease progression. Blood inflammatory markers associated with physiological processes will be quantified (such as TNFα, IL-6, IL-10, IFN-γ, C-reactive protein (CRP), Zonulin (Haptoglobulin), and IL-1β). It is hypothesised that pectin supplementation over 6 weeks will reduce circulating inflammatory biomarkers compared with placebo supplementation. The secondary aims of the study are to assess changes in anthropometric measures; to evaluate changes in general metabolic indicators, including fasting blood glucose and blood-based biomarkers relevant to MASLD activity and fibrosis, including liver-associated enzymes (alanine aminotransferase (ALT), aspartate aminotransferase (AST), gamma-glutamyl transferase (GGT), and alkaline phosphatase (ALP)), bilirubin levels, lipid profiles, and platelet counts; to explore alterations in gut microbiome composition; to examine modifications in non-invasive physiological liver assessments, including fat content and stiffness; to observe changes in hepatic and abdominal organ fat using magnetic resonance imaging (MRI) to investigate changes in gut permeability; and to investigate the presence of genetic variants associated with development of MASLD.

## Materials and methods

### Study design and setting

**Fig 1:**
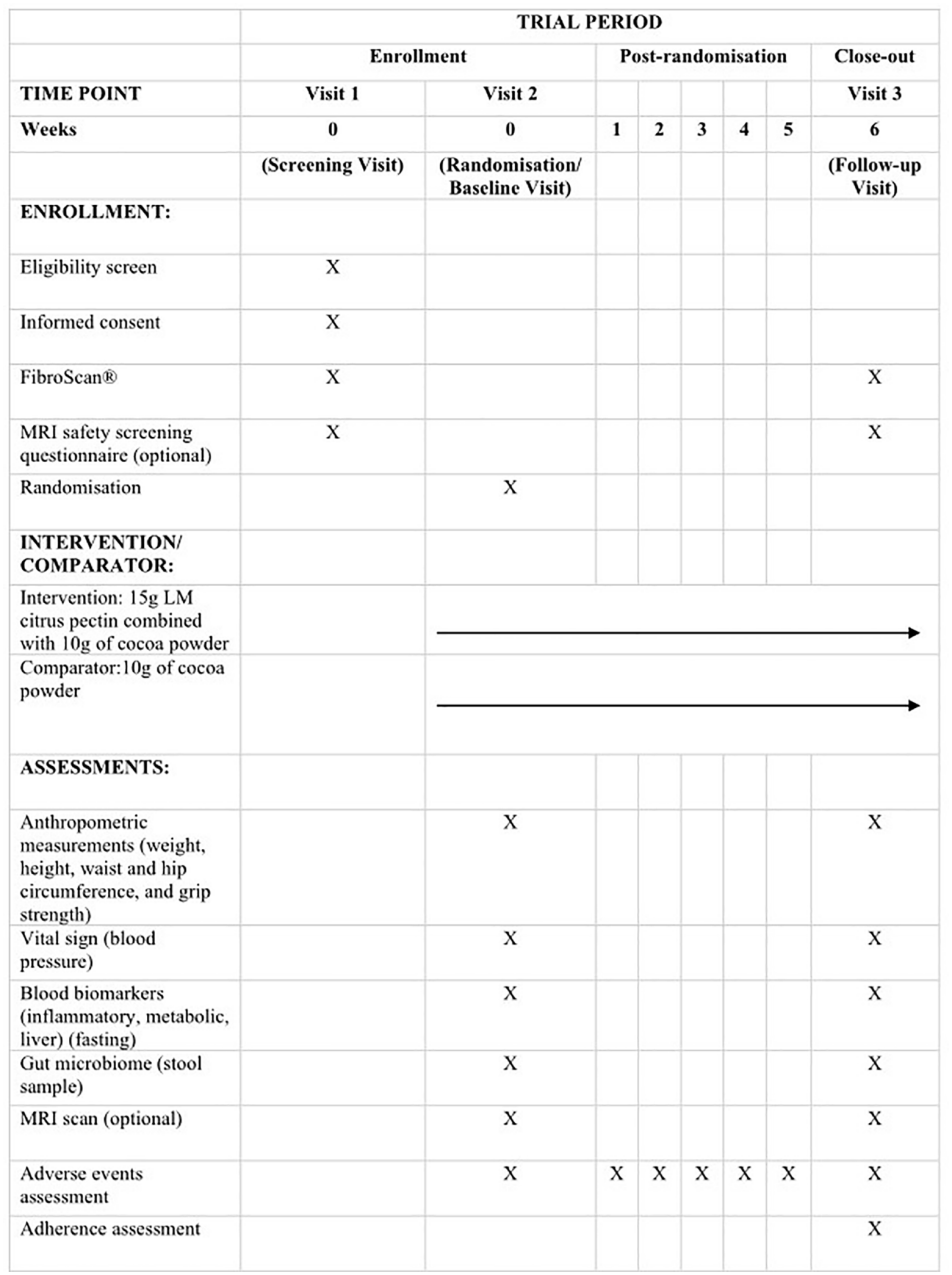
SPIRIT Schedule of Enrolment, Interventions, and Assessments. This figure illustrates the timing of participant enrolment, randomisation, intervention delivery, and outcome assessments across the study period. Abbreviations: LM citrus pectin, Low-methoxyl pectin; MRI, magnetic resonance imaging..

This study is a single-centre, randomised, double-blind, placebo-controlled, parallel-group dietary intervention to be conducted over 24 months at Nottingham University Hospitals NHS Trust (NUH), in collaboration with the University of Nottingham. The overall protocol design follows the Standard Protocol Items: Recommendations for Intervention Trials (SPIRIT) guidelines [16] (S1 Table and Fig 1).

### Participant screening and recruitment

Recruitment and clinical visits will take place at the Queens Medical Centre, Nottingham University Hospitals NHS Trust. Samples will be processed and stored at the University of Nottingham. Laboratory analyses will be at the University of Nottingham or by contracted service providers. MRI scans are optional and will be undertaken at the Sir Peter Mansfield Imaging Centre (SPMIC) at the University of Nottingham.

Participants will be identified and recruited in a secondary care setting at the Queen’s Medical Centre, Nottingham. Recruitment will occur via advertisements (posters displayed in relevant clinical areas and University premises, and via websites and social media), direct approach in hepatology outpatient clinics by members of the usual-care team, and via invitation of individuals with a prior MASLD diagnosis who previously participated in studies led by the Chief Investigator and consented to future contact. Interested individuals will attend a screening visit to confirm eligibility and obtain baseline measurements.

The study is conducted in accordance with the International Conference on Harmonisation and Good Clinical Practice (ICH-GCP) guidelines [17]. Written informed consent will be obtained by trained research staff before any study procedure is undertaken. If a participant chooses to withdraw, they will be advised that data collected before withdrawal may not be removed from the dataset, and consent will be obtained to retain and include these data in the final analysis where appropriate. If a participant withdraws or is unable to complete the intervention after randomisation, an additional participant will be recruited and allocated to the same intervention group to maintain the 1:1 allocation ratio between the two study arms. Recruitment will continue until a total of 30 participants have completed all study procedures.

15 healthy volunteers will be recruited and undergo 2 scans 6 weeks apart (without intervention). This data will be used to establish the repeatability of our MRI protocol [18], in particular, the measurement of small intestinal wall thickness using a new method. They will be used as a comparator for the patient cohort at baseline. The interventional study aims to recruit 30 participants with MASLD. The inclusion and exclusion criteria are listed in Table 1 below.

**Table 1:** Eligibility criteria.

Category	Eligibility criteria
**Inclusion criteria for healthy volunteers undergoing MRI**	Able and willing to provide informed consent.
	Aged ≥18 years.
	CAP < 250 dB/m and VCTE <8 kPa by FibroScan® within the previous 6 months.
**Inclusion criteria for the interventional study**	Patients with a clinical diagnosis of MASLD, having an assessment suggesting that liver fat > 5% (e.g., histological evidence and/or Transient Elastography using CAP – FibroScan® in the past month and/or liver imaging (such as CT, US or MRI).
	Able and willing to provide informed consent.
	Aged ≥18 years.
	Body mass index (BMI) between 18.5 and 39.9 kg/m².
	Stable body weight (≤3 kg change within the previous 3 months).
	For participants with diabetes: HbA1c < 7.0% (<53 mmol/mol) [19].
	Able to undergo FibroScan® assessment.
**Exclusion criteria for the interventional study**	Have an allergy to soya, milk or chocolate.
	Have an allergy to pectin.
	Participants on a vegan diet.
	Have eating disorders or difficulties or gastrointestinal conditions, e.g., malabsorptive conditions such as coeliac, IBS, IBD or gastroparesis.
	Have chronic malnutrition condition.
	History of major surgery, which potentially limits participation or completion of the study.
	History of previous intestinal surgery known to affect food intake or digestive function, including bariatric surgery.
	Use of antibiotics, antifungal medications, probiotics or prebiotics 90 days before the start of the study.
	Are taking the following medications: immunosuppressants, amiodarone and/or perhexiline.
	Are currently following or anticipated to commence a specialised commercially available weight loss diet and/or program, or concomitant use of any weight loss medication or herbal weight loss products.
	History of side effects towards probiotics or prebiotics.
	History or current psychiatric illness.
	History or current neurological condition (e.g., epilepsy).
	Participants with other liver abnormalities.
	Evidence of monogenic metabolism diseases such as Lysosomal acid lipase deficiency, Wilson disease, Hypobetalipoproteinemia, or inborn errors of metabolism.
	Have had a weight change exceeding 3 kg within 3 months.
	Uncontrolled diabetes, active malignancy, or chronic infections.
	Having symptoms of active infection.
	Excessive alcohol intake is defined as self-reported intakes greater than 21 units per week in men, and 14 units per week in women.
	Participants who are pregnant, breastfeeding, or actively planning pregnancy will be excluded from the study.
	Participation in any other trial in the last 3 months.
**MRI exclusion criteria**	Contraindications for MRI scanning: having pacemakers, defibrillators, neurostimulators, prohibited medical implants, and foreign bodies (e.g., bullets, shrapnel, metal slivers), history of metallic foreign body in eye(s) and penetrating eye injury that could present a risk during an MRI scan.
	Difficulty breathing or inability to lie flat, as well as conditions that could worsen under stress (such as anxiety or panic disorders, claustrophobia, uncontrolled hypertension, or seizure disorders) severe enough to prevent undergoing an MRI.
	Contraindications to Hyoscine butylbromide: Previous adverse drug reaction (allergic, hypersensitivity or other), Angle-closure glaucoma, tachycardia, ischemic heart disease, myasthenia gravis, prostatic enlargement with urinary retention requiring catheterisation, mechanical stenosis in the gastrointestinal tract, paralytic or obstructive ileus, hypotension, cardiac disease, recent heart attack or any arrhythmias, thyrotoxicosis, gastro-esophageal reflux disease, hiatus hernia, ulcerative colitis.

Abbreviations: BMI, body mass index; CAP, controlled attenuation parameter; CT, computed tomography; HbA1c, Hemoglobin A1C; IBD, inflammatory bowel disease; IBS, irritable bowel syndrome; MASLD, metabolic dysfunction–associated steatotic liver disease; MRI, magnetic resonance imaging; US, ultrasound; VCTE, vibration-controlled transient elastography

### Randomisation

Upon enrolment, participants with MASLD will be randomly assigned to the intervention or placebo arm in a 1:1 ratio using a computer-generated random allocation sequence created by an independent researcher not involved in participant recruitment or assessment.

Allocation concealment will be ensured through a secure, independent web-based randomisation service (www.sealedenvelope.com) inaccessible to investigators responsible for enrolment. Participants, investigators, and outcome assessors will remain blinded to group allocation until completion of data analysis, unless unblinding is required for safety reasons.

Blinding will be achieved through the use of visually and sensorially matched supplements, with identical packaging, labelling, and preparation procedures for both intervention and placebo products.

Unblinding will only occur if required for participant safety in the event of a medical emergency. In such cases, allocation will be revealed by an authorised member of the study team via the randomisation system, and the reason for unblinding will be documented.

### Intervention and comparator

Participants in the intervention group will receive a daily supplement of 15g of LM citrus pectin with a degree of methyl-esterification (DM) below 10% (DM = 9.5, BOC Sciences, United States) combined with 10g of cocoa powder (Nesquik®, NESTLE UK LTD, United Kingdom) added as flavour. The control group will receive a placebo, a daily supplement of 10g of cocoa powder.

The use of a cocoa powder–based placebo allows for appropriate matching of taste, appearance, and mode of administration between groups, thereby minimising performance and expectation bias.

The placebo formulation was designed to be non-fermentable and metabolically inert, in order to isolate the specific effects of LM pectin on gut microbiota and inflammatory pathways. Unlike fermentable fibres, inclusion of additional fibre in the placebo arm was intentionally avoided to prevent confounding effects on microbial fermentation, short-chain fatty acid production, and immune modulation.

The selected intervention dose in the present study was informed by previous human supplementation studies demonstrating acceptable tolerability and measurable biological effects at doses ranging from 15–20 g/day of LM pectin [15]. This dose range was also reviewed in consultation with dietetic specialists during protocol development and was considered appropriate within the context of habitual dietary fibre intakes, which are typically below recommended intake levels in the general population. Current nutritional guidance recommends total daily dietary fibre intakes of approximately 25–30 g/day in adults [20].

Study supplements will be packaged identically to ensure concealment. Each participant will receive daily portions of supplements in separate food-safe zip-lock bags to be consumed over the next 42 days. They will be advised to pour 250–300 ml of water into a shaker bottle, then add the entire supplement powder portion and shake the bottle until the contents reach a shake-drink consistency. Participants will be advised to consume this as soon as possible.

Adherence will be monitored using a daily supplement tracker provided to each participant, in which participants record the number of days they consume the supplement. Compliance will also be assessed by returning and counting unused supplement bags at the end of the intervention period. Adherence will be considered acceptable if ≥80% of the prescribed supplementation is consumed.

At baseline, participants will complete study case report forms (CRFs) including questions relating to habitual dietary fibre intake and current probiotic use. These data will be used to characterise baseline dietary patterns and identify substantial dietary changes during the intervention period that may influence microbiome or inflammatory outcomes. Participants will be asked to maintain their usual diet and physical-activity levels throughout the trial and to avoid introducing new fibre supplements or probiotics.

### Study procedures

Consented healthy volunteers will attend 2 study visits for MRI scans at baseline and after 6 weeks. No other tests will be performed.

For patients with MASLD, after screening, each participant will attend two study visits. At Baseline (Week 0), there is an anthropometric assessment, vital signs, fasting blood sampling, stool collection, FibroScan®, and an optional MRI scan. At follow-up (Week 6), all baseline assessments are repeated (Fig 1).

MRI assessments are optional and are included as mechanistic sub-study due to participant burden, contraindications, and scanner availability constraints, while primary outcomes and other secondary outcomes are collected in all participants.

To support participant retention and minimise missing data, participants will receive regular study reminders via email or telephone where appropriate. Flexible scheduling of study visits will be offered where feasible, and reimbursement for travel expenses will be provided in accordance with institutional policy. Participants will also receive clear written and verbal instructions regarding supplement administration and study procedures to support adherence throughout the intervention period.

### Outcomes

#### Primary outcomes.

The primary endpoint is the change in circulating inflammatory biomarkers (e.g. TNFα, IL-6, IL-10, IFNᵞ, CRP, Zonulin (Haptoglobulin), IL-1β) between baseline and follow-up. This endpoint was chosen based on a previous pectin pilot study that was conducted on healthy volunteers and which found significant differences in 4 proinflammatory markers [15].

#### Secondary outcomes.

Secondary endpoints include changes in anthropometric measurements and fasting blood glucose. Additional secondary measures comprise alterations in blood-based biomarkers relevant to MASLD activity and fibrosis, including full-length and cleaved cytokeratin-18, NIS2 + ™, YKL-40, and routine liver-associated enzymes (ALT, AST, GGT, ALP), bilirubin, lipid profile, and platelet count.

Microbiome-related secondary outcomes include changes in gut microbial composition and diversity assessed by 16S rRNA sequencing. Imaging-based secondary endpoints include changes in hepatic steatosis and stiffness measured by FibroScan® CAP and in liver stiffness measured by vibration-controlled transient elastography (VCTE). In a subset of participants, MRI will assess hepatic fat and subcutaneous and visceral adipose tissue using mDixon, hepatic fibrosis and inflammation using liver T1, T2, diffusion and MR elastography, and intestinal permeability using T2 measures.

Outcome selection was informed by current understanding of MASLD pathophysiology and prioritised clinically relevant, patient-centred, and non-invasive measures where feasible. Given the exploratory and mechanistic nature of this study, outcomes were selected to evaluate inflammatory, metabolic, hepatic, and gut microbiome-related pathways implicated in MASLD progression while minimising participant burden. The study is intended to generate preliminary mechanistic and feasibility data to inform future larger-scale clinical trials, and the development of core outcome sets for nutritional interventions in MASLD.

### Outcome assessments

All outcome measures will be assessed at baseline (week 0, before intervention) and at post-intervention follow-up (week 6), unless otherwise specified. Participants will attend study visits in the morning following a 6-hour overnight fast. Due to FibroScan® requirements, participants will be advised to avoid alcohol for at least 24 hours before the assessment and to stop drinking water 2 hours before the scan visit.

#### Anthropometry.

Anthropometric measurements will be performed using calibrated equipment and standardised procedures. Body weight will be recorded with participants wearing light clothing and no shoes. Height will be measured at baseline using a wall-mounted stadiometer, and BMI will be calculated as kg/m². Waist circumference will be measured at the level of the umbilicus (belly button) using a non-elastic measuring tape. Hip circumference will be measured at the widest point over the buttocks. Blood pressure will be measured in the seated position after 5 min of rest, and grip strength will be measured using the Takei 5401 Hand Grip Digital Dynamometer.

#### Blood biomarkers.

To measure circulating inflammatory biomarkers, fasting blood glucose, blood-based biomarkers relevant to MASLD activity and fibrosis, liver enzymes, bilirubin, lipid profile, and platelet count, 20 ml of fasting venous blood samples will be collected using suitable additives or coagulated for serum, from an antecubital vein, processed within 1 hour of collection, centrifuged at 2000 × g for 10 min at 4 °C, aliquoted, and stored at −80°C until analysis. Whole blood preserved with EDTA collected for genetic analysis will be stored at −80°C, and DNA will be extracted using the Flexigene kit (Qiagen, UK). Biomarkers will be determined using commercial kits (e.g., ELISA), published methods [21] or by commercial service providers.

#### Stool samples.

Stool samples will be collected at home 24 hours before each visit and will be stored in aliquots at −80°C until analysis. Microbial DNA will be extracted, and 16S rRNA gene sequencing will be performed to determine the bacterial species present based on their characteristic DNA signatures.

#### Non-invasive hepatic assessments.

Non-invasive hepatic assessments will be performed in all participants for the interventional study using FibroScan® (Echosens, Paris, France). CAP will quantify hepatic steatosis, and liver stiffness measurement obtained via VCTE will assess fibrosis.

#### Magnetic-resonance imaging (MRI).

MRI scans are optional. If a participant consents to taking part in the MRI sub-study, then they will be asked to fast for at least 6 hours prior to each visit. The MRI data will be collected on a Philips 3.0T Ingenia wide-bore MR scanner. The MR protocol comprises a 6-echo mDixon (3D multi-echo gradient echo (GRE) scan with complete coverage of the liver collected. This 6-echo mDIXON will be analysed to provide a fat fraction map correcting for T2* decay, eddy currents, and B0 field inhomogeneities. This will be used to assess hepatic fat fraction.

This will be followed by quantitative respiratory-triggered liver T1, T2 and DWI mapping as well as MR Elastography (MRE) to study fibrosis and inflammation. T1 mapping will be collected using an inversion recovery (IR) fat-suppressed spin-echo echo planar imaging scheme with data fit to an inversion recovery [22], and a dual-echo 5-3-3 MOLLI scheme which, after water–fat separation, will be fit to the Look-Locker model [23]. Liver T2 mapping data will be collected using a multi-echo spin echo sequence utilising extended phase graph (EPG) algorithms to generate accurate T2 maps [24], while diffusion weighted imaging (DWI) will be collected at 13 b-values to generate an Apparent Diffusion Coefficient (ADC) map. MRE will be collected using the QIBA protocol with a SE-EPI readout [25]. A 3-stack 2-echo mDIXON with complete coverage of the abdomen will be collected to quantify the volume of visceral adipose tissue (VAT) and abdominal subcutaneous adipose tissue (SAT). These liver scans will take approximately 35–40 minutes to acquire.

Then, the study participant will then be taken out of the scanner, and a medical doctor will cannulate the participant to administer hyoscine butylbromide later. The participants will be asked to drink the oral contrast, which consists of 1 litre of 2.5% Mannitol and 0.2% Locust Bean Gum (w/w) prepared on the morning of the scan. The participant will be asked to drink the oral contrast gradually over 40 minutes, this allows the lining of the gut to be distinguished from its contents. After, the participant will be put back into the scanner. The medical doctor will then administer 2 doses of hyoscine butylbromide, 20 mg/ml each through the cannula to stop the bowel from moving to ensure clear pictures of the bowel wall [18]. T2 MRI measurements of the gut wall will then be collected in multiple locations to measure gut permeability followed by a B1 map of the abdomen to be used in the T2 fit.

MRI outcomes are not required for primary endpoint evaluation and are included to provide additional mechanistic insight into hepatic and intestinal physiology.

### Safety considerations

This study is not aimed at primarily assessing safety, because given the dietary nature of the intervention, no serious adverse events are anticipated. The investigational product, pectin, is a widely consumed dietary fibre, and the expected effects are limited to mild gastrointestinal symptoms, including bloating, abdominal discomfort, flatulence, or diarrhoea, that may occur particularly among individuals with low habitual fibre intake. Participants will therefore be monitored throughout the intervention period for gastrointestinal symptoms and tolerability.

To minimise risk, participants with pre-existing gastrointestinal conditions (including inflammatory bowel disease, irritable bowel syndrome, or prior gastrointestinal resection surgery), known food allergies or sensitivities to study components will be excluded.

Pregnant and breastfeeding women will be excluded from the study because there is limited data regarding the safety of pectin consumption during pregnancy and breastfeeding.

For participants undergoing MRI assessments, additional safety measures will be implemented. Hyoscine butylbromide, known transient effects (e.g., blurred vision, tachycardia) will be monitored, and participants will remain under observation until symptom resolution. Rare adverse reactions, including skin reactions, dry mouth, dyshidrosis, and in extremely rare cases, angle-closure glaucoma, will be managed by a medically qualified member of the research team present during scanning. Oral contrast (mannitol 2.5% with locust bean gum 0.2%) may cause mild gastrointestinal effects, including flatulence or diarrhoea, which will be monitored during study visits. All adverse events will be recorded, categorised by severity and relatedness to the intervention, and reported in accordance with Good Clinical Practice guidelines.

### Stopping rules and discontinuation

If a participant develops a severe adverse reaction following consumption of the supplement (i.e., severe bloating amongst those who have a low habitual intake of dietary fibre), they will be asked to contact the study team immediately. Based on the severity of the event, the study team may advise the participant to continue or withdraw from the study.

Moreover, participants who agree to have MRI scans will be carefully monitored during the scan, and if their medical or physiological status alters rapidly, then it may be necessary to stop scanning based on the radiologist’s advice.

Participants may withdraw from the study at any point. Significant non-compliance with the study schedule may lead to a participant being withdrawn from the study.

The study team will try to replace withdrawn participants.

### Data management

#### Data collection and management system.

Study data will be collected and managed using REDCap (Research Electronic Data Capture), a secure, web-based clinical data management system hosted by the University of Nottingham. Each participant will be assigned a unique trial identification number at randomisation, which will be used on all case report forms (CRFs), biological samples, and study documents. Identifiable information will be stored separately from research data and accessible only to authorised personnel.

Electronic CRFs will capture participant demographics, medical history, anthropometric measurements, imaging results, and laboratory outcomes. All study data will be handled in accordance with the ICH-GCP guidelines [17] and the UK Data Protection Act 2018 [26].

#### Data security, access, and quality control.

Access to study data will be restricted through role-based permissions, with individual user authentication. All electronic data will be stored on secure, password-protected University of Nottingham servers with encryption and audit trails to ensure traceability of data entry and modifications. Paper records will be stored in locked cabinets within secure research facilities.

Data quality will be ensured through predefined validation rules within REDCap, regular monitoring, and source data verification. A proportion of CRFs will be cross-checked against source documents to ensure accuracy and completeness. Any data modifications will be documented with a full audit trail.

#### Data storage, backup, and archiving.

All electronic data will be regularly backed up to secure local and remote servers in an encrypted format. MRI data will be downloaded from the 3T University of Nottingham MRI machines and stored on secure institutional drives. Study data will be retained for a minimum of 7 years in accordance with regulatory requirements and institutional policy. At study completion, anonymised datasets will be prepared for analysis and potential sharing in line with ethical approvals.

#### Confidentiality and data protection.

Participant confidentiality will be ensured through the use of unique coded identifiers, with personally identifiable information stored separately from research data. Access to identifiable data will be restricted to authorised study personnel and relevant regulatory authorities where required for monitoring, audits, and inspections. All data used for analysis and dissemination will be fully anonymised.

#### Compliance.

No interim data analyses are planned, and given the low-risk dietary nature of the intervention, the study team will not need a data monitoring committee nor the oversight of a formal study steering committee.

### Sample size and statistical analysis

The sample size for the interventional study was calculated a priori based on a previous pilot study in healthy volunteers, in which supplementation with LM pectin significantly reduced pro-inflammatory markers (TNFα, IL-1β, IL-6, INF-γ) compared to control (n = 14 per group) [15]. Using the observed mean differences and standard deviations for these primary outcomes, a sample size of 14 participants per arm provides approximately 80% power to detect a similar effect at a two-sided independent samples t-test (difference between two means), assuming equal allocation between groups (1:1) with a significance level of α = 0.05. To allow for potential dropouts or missing data, 15 participants will be recruited per arm.

The sample size for the MRI sub-study was calculated based on previously published quantitative MRI research evaluating changes in small bowel permeability following indomethacin provocation in healthy volunteers. That study reported a significant increase in small bowel wall T2 (mean ± standard deviations (SD): 0.115 ± 0.063 s vs. 0.070 ± 0.036 s) [18]. As the reported standard deviations differed between groups, unequal variance assumptions were applied during sample size estimation. These data correspond to an estimated effect size of approximately 0.88. Based on a two-sided significance level of 0.05 and 80% statistical power, approximately 11 participants per group are required to detect a difference of this magnitude. Accordingly, 22 participants with MASLD will be included in the MRI sub-study, with 11 allocated to the LM pectin intervention and 11 to the control group. In addition, to support the reliability and reproducibility of the quantitative MRI measurements used in this study, 15 healthy volunteers will be recruited, undergoing two MRI scans separated by six weeks without any intervention. The repeatability data will be used to determine the smallest detectable change in MRI measures for comparison with changes measured in the intervention study. Allowance was made for potential motion-related artefacts and non-analysable datasets, which are commonly reported in gastrointestinal MRI studies.

Intestinal wall T2 repeatability measures will extend previous work from Alyami et al [27] by collecting these data in more than 1 slice and incorporate the B1 mapping into the T2 fit.

Data input, cleaning, and analysis will be conducted using statistical software packages, including GraphPad Prism (version 11.0.0; GraphPad Software, Boston, MA, USA) or IBM^®^ SPSS^®^ (version 29.0.1.0; IBM Corp, Armonk, New York) and R software (version 4.4.2; R Foundation for Statistical Computing, Vienna, Austria). Descriptive statistics will be reported as means ± standard deviations (SD) or medians with interquartile ranges (IQR) for continuous variables, and as frequencies and percentages for categorical variables. Before analysis, data will be checked for normality using the Shapiro-Wilk test. Standard parametric and non-parametric tests (where assumptions of normality are violated) will be used to assess the significance of changes compared to baseline on both the pectin arm and the placebo arm. Mixed-effects linear regression models with a random intercept for subjects will be used to assess the intervention effect of pectin compared to placebo, with the primary focus on the time (baseline vs. follow-up) × group (pectin vs. placebo) interaction to evaluate differential changes over time. Models will adjust for baseline values of the inflammatory markers and relevant covariates (age, sex, ethnicity, BMI), provided they are associated with the outcome or imbalanced between groups. The normality of inflammatory markers and model residuals will be assessed using graphical methods (e.g., histograms, Q-Q plots) and, if needed, statistical tests (e.g., Shapiro-Wilk). If normality is violated, appropriate transformations (e.g., logarithmic) will be applied to meet model assumptions. Where substantial non-normality persists despite transformation, additional sensitivity analyses using appropriate non-parametric or rank-based longitudinal methods will be considered. For exploratory analysis, change scores may be analysed using linear regression models or non-parametric tests, depending on their distribution. Model assumptions, including homoscedasticity and residual normality, will be assessed using standard diagnostic plots.

Given the number of secondary and exploratory outcomes assessed, findings from these analyses will be interpreted cautiously. To reduce the risk of type I error associated with multiple testing, false discovery rate (FDR) correction methods will be applied where appropriate. A p-value < 0.05 will be considered statistically significant for primary analyses.

Missing data will be handled using appropriate methods depending on the extent and mechanism of missingness, including mixed-effects modelling under the missing-at-random (MAR) assumption and sensitivity analyses where appropriate. The MAR assumption is considered reasonable given the short intervention duration, frequent participant contact, and planned retention procedures. To minimise missing data and improve adherence, participants will receive regular reminder communications, flexible visit scheduling where feasible, and reimbursement for study participation.

### Ethics

The study was approved by the North West – Preston Research Ethics Committee at the United Kingdom (Approval No:25/NW/0062).

### Protocol amendments

Any protocol amendments will be communicated to relevant regulatory bodies, trial registries, and investigators, and updated in the trial registry where applicable.

### Publication and dissemination policy

The results of this study will be reported in accordance with established reporting guidelines and disseminated through multiple channels. Study findings will be submitted for publication in peer-reviewed scientific journals and presented at national and international conferences. Outputs from the study will also contribute to academic theses and student research outputs, where applicable.

Summary results will be posted on ClinicalTrials.gov following completion of data analysis. Reports will be provided to the study funder in line with funding requirements. All publications will preserve participant confidentiality, and no individual will be identifiable in any disseminated material.

Where appropriate, fully anonymised datasets underlying the results reported in this study will be made available upon reasonable request to the corresponding author, in accordance with ethical approvals and participant consent, to support transparency and reproducibility of findings. In addition, lay summaries of the results will be prepared and shared via institutional platforms, including the NIHR Nottingham Biomedical Research Centre website and associated University and NHS channels. Participants who have consented to future contact will be provided with a summary of the study findings.

### Trial status and timeline

This manuscript is based on protocol version 1.2 (dated 30 June 2025). The trial was prospectively registered on ClinicalTrials.gov (Identifier: NCT07093346) on 30 July 2025. Registration occurred after initiation of participant recruitment due to administrative delays related to completion of institutional approvals and registry documentation processes. The study protocol, primary and secondary outcomes, and statistical analysis plan were predefined before enrolment of the first participant. The authors confirm that all ongoing and related trials for this intervention are registered.

Participant recruitment commenced on 30 June 2025 and is ongoing at the time of manuscript submission. Recruitment is anticipated to be completed by 31 December 2026.

Data collection will occur at baseline and post-intervention follow-up and is expected to be completed following the final participant visit. Data cleaning and statistical analyses are scheduled to take place between January and March 2027.

The study results are expected to be available following completion of the analysis phase and will be disseminated in accordance with the study’s publication and dissemination plan.

### Patient and public involvement

Patients and the public contributed to the design of this study. The NIHR Nottingham Biomedical Research Centre Digestive Diseases Patient Advisory Group provided input on the study concept, highlighting the need for practical and acceptable interventions for metabolic dysfunction–associated steatotic liver disease. Feedback from focus groups informed the development of study procedures and participant materials to enhance clarity and accessibility.

Insights from individuals who had participated in previous dietary intervention studies indicated challenges in maintaining complex dietary changes and a preference for simpler approaches, which informed the choice of a supplementation-based intervention.

Patient engagement activities also contributed to identifying research priorities and refining the study design. Ongoing involvement is planned to support dissemination, including the development of lay summaries and communication of findings to participants and the wider public.

## Discussion

This trial is designed to investigate the effects of six-week LM pectin supplementation in adults with MASLD. It will evaluate whether LM pectin can modify systemic inflammation, hepatic characteristics, and gut-microbiota composition compared with placebo. To our knowledge, this is the first randomised controlled trial in humans specifically targeting the effects of LM in patients with MASLD. The study has been designed as an exploratory mechanistic trial intended to generate preliminary clinical and biological evidence that may inform future larger-scale efficacy studies and the development of core outcome sets for nutritional interventions in MASLD.

This interventional study aims to recruit 30 participants with MASLD, of whom 22 will participate in the MRI sub-study. Test-retest MRI repeatability measures will be determined in 15 healthy volunteers by comparing baseline and 6-week scans. Comparisons will also be made between the healthy volunteer and patient MRI measures at baseline.

The rationale for selecting LM pectin is based on its physicochemical and biological properties. Pectin forms viscous gels in the intestine, slows nutrient absorption, and serves as a fermentable substrate for beneficial microbiota that generate short-chain fatty acids (SCFAs). SCFAs can play an important role in lipid and glucose homeostasis by entering the peripheral circulation through the portal vein and then acting on the liver and peripheral tissues. Moreover, inside the intestine, short-chain fatty acids can enhance the secretion of peptide YY (PYY) and Glucagon-like peptide 1 (GLP-1) by enteroendocrine cells, and both peptides are responsible for slowing down the intestinal transit and suppressing food intake.

Both in vivo and in vitro studies have shown that pectin can increase SCFAs. Moreover, research has indicated that pectin can strengthen gut barrier function and reduce permeability by increasing the amount of SCFAs that can promote the production of mucus in the intestines of rodents and improve the building of tight junctions. Studies showed that LM pectin produced a higher amount of SCFAs compared to HM pectin [13,28,29]. LM pectin helps reinforce the structure and function of tight junctions that regulate the selective permeability of the gut, allowing nutrients and water to pass while blocking harmful substances. Studies suggest that LM pectin increases the expression of proteins involved in tight junction integrity, such as occludin. This reinforcement reduces the likelihood of molecules leaking through the intestinal barrier [30].

Research using human and animal models has demonstrated that pectin has potential anti-inflammatory effects by interacting with Toll-like receptors (TLRs). TLRs play a crucial role in the progression of liver inflammation and steatosis. TLR2 and TLR4 are the most common TLRs associated with metabolic inflammation. TLR2 up-regulation was reported in many NAFLD animal models, as well as leading to the development of MASH in the liver of E3 rats. In addition, anti-TLR2 antibody reduces hepatic injury, inflammation, fibrosis and steatosis in rats with obesity-related metabolic disorder via regulation of MAPK and NF-κB pathways [31,32]. Pectin can directly bind to TLRs and inhibit their activity based on its degree of esterification; the higher the degree of methyl-esterification, the lower the binding to TLRs. LM pectin was able to strongly inhibit TLR2–1-induced IL-6 secretion in human macrophages [33]. In in vitro studies that utilised mouse macrophages and human dendritic cells, LM pectin showed an anti-inflammatory response by directly binding to TLR2–TLR1 receptors. This effect was independent of the effects due to SCFAs produced by the gut microbiota [34]. By combining validated non-invasive measures of hepatic steatosis (FibroScan® and MRI), biomarker profiling, and microbiome sequencing, this study aims to integrate metabolic and microbial endpoints to explore pectin’s multifaceted actions.

This trial combines several methodological strengths designed to maximise internal validity and mechanistic insight: the randomised, double-blind, placebo-controlled structure minimises bias and allows causal inference about pectin’s effects, using FibroScan® offers a quantitative, non-invasive assessment of steatosis and fibrosis, suitable for repeated measures, parallel analyses of circulating cytokines, metabolic markers, and 16S rRNA sequencing will provide an important view of diet-microbiome–liver interactions, use of purified LM pectin ensures reproducibility and avoids confounding by other fruit-derived bioactive substances, and the supplement is safe, affordable, and easily implementable within general dietary practice. Furthermore, the inclusion of clinically relevant non-invasive outcome measures aligns with increasing emphasis on patient-centred monitoring approaches in MASLD research and clinical care. If pectin demonstrates measurable improvements in inflammatory or hepatic outcomes, the findings could justify larger trials assessing clinical endpoints such as histological response.

Several limitations must be acknowledged. First, the relatively small sample size since the trial is exploratory and intended primarily to establish feasibility and mechanistic signals. Second, the six-week duration of the intervention may be insufficient to observe significant changes in hepatic fat content, though shorter-term improvements in inflammation or gut-barrier function might be observable. Third, dietary adherence relies on self-reporting and sachet counts, which may introduce reporting bias; however, this is mitigated through frequent contact. Finally, MRI is optional rather than mandatory due to logistical considerations, participant burden, scanner availability, and MRI contraindications; thus, this data will be available only in a subset of participants. Nevertheless, all participants will undergo FibroScan®, ensuring complete quantitative data for liver stiffness and steatosis.

## Supporting information

S1 Table SPIRIT 2025 editable checklist: Recommended items to address in a clinical trial protocol. (DOCX)

S1 File PROTOCOL V1.1 11032025. (DOCX)

S2 File PROTOCOL V1.2 30062025. (DOCX)
