# Supplementary material for: The impact of pectin supplementation on systemic inflammation pathways, gut microbiome, and metabolic health in patients with Metabolic Dysfunction-Associated Steatotic Liver Disease (MASLD): A study protocol for a randomised controlled trial
Source: PLoS One. 2026 Jul 13;21(7):e0352397. doi: 10.1371/journal.pone.0352397 (PMC13362096; doi:10.1371/journal.pone.0352397)
Supplement: S1 File — (DOCX) [file pone.0352397.s002.docx]

**The Impact of Pectin Supplementation on Systematic Inflammation Pathway, Gut Microbiome, and Metabolic Health in Patients with Metabolic Dysfunction-Associated Steatotic Liver Disease (MASLD): A Randomised, Placebo-Controlled, Dietary Intervention Study**

**Final Version 1.1**

**11 March 2025**

**Short title:** *Pectin Supplementation and MASLD*

**Acronym:** PEC-MASLD

**Trial Registration:** [www.clinicaltrials.gov](http://www.clinicaltrials.gov)

**ISRCTN:** *if appropriate*

**IRAS Project ID:** *351797*

**Trial Sponsor:** University of Nottingham

**Sponsor reference:** *24076*

**Funding Source:** NIHR Nottingham Biomedical Research Centre

TRIAL / STUDY PERSONNEL AND CONTACT DETAILS

**Sponsor:** University of Nottingham

Contact name Ali Alshukry

Head of Research Integrity, Governance & Compliance

University of Nottingham

E-floor, Yang Fujia Building

Jubilee Campus

Wollaton Road

Nottingham

NG8 1BB

**Chief investigator:** **Professor Guruprasad P Aithal**

Nottingham Digestive Diseases Centre & NIHR

Nottingham Biomedical Research Centre,

Nottingham, University Hospitals NHS Trust and the

University of Nottingham, Queens Medical Centre

Nottingham, NG7 2UH

UoN job title: Professor of Hepatology

Add the name and contact details of the medical expert if different to the CI.

**Co-investigators:**

**Dr Jane Grove**

Associate Professor

Nottingham Digestive Diseases Centre & NIHR

Nottingham Biomedical Research Centre,

Nottingham, University Hospitals NHS Trust and the

University of Nottingham, Queens Medical Centre

Nottingham, NG7 2UH

**Noor Al-Tameemi**

PhD Student

Nottingham Biomedical Research Centre,

Nottingham, University Hospitals NHS Trust and the

University of Nottingham, Queens Medical Centre

Nottingham, NG7 2UH

Prof Penny Gowland

Professor of Physics (Magnetic Resonance)

Sir Peter Mansfield Imaging Centre

School of Physics and Astronomy

University of Nottingham

University Park

Nottingham

NG7 2RD

Phone: 01159514754

Fax: 01159515166

Email:[Penny.Gowland@nottingham.ac.uk](mailto:Penny.Gowland@nottingham.ac.uk)

Dr Caroline Hoad

Senior Research Fellow

Sir Peter Mansfield Imaging Centre

School of Physics and Astronomy

University of Nottingham

University Park

Nottingham

NG7 2RD

Professor Susan Francis

Professor of Physics (Magnetic Resonance)

Sir Peter Mansfield Imaging Centre

School of Physics and Astronomy

University of Nottingham

University Park

Nottingham

NG7 2RD

**Trial / Study Statistician:** **Professor Ana M Valdes**

Professor in Genetic and Molecular Epidemiology

Surgery, Trauma and MSK Ageing

Nottingham Biomedical Research Centre,

University of Nottingham, Room A28a, Clinical Sciences Building, City Hospital Nottingham

Nottingham, NG5 1PB

**Trial / Study Coordinating Centre:** Nottingham Digestive Diseases Centre,

University of Nottingham, Queens Medical Centre,

Nottingham, NG7 2UH

SYNOPSIS

| Title | The Effect of Pectin Supplementation on Patients with Metabolic Dysfunction-Associated Steatotic Liver Disease (MASLD): A Randomised Placebo-Controlled Dietary Intervention Study |
| --- | --- |
| Acronym | PEC-MASLD |
| Short title | Pectin Supplementation and MASLD |
| Chief Investigator | Professor Guruprasad P Aithal |
| Objectives | To investigate the effects of daily pectin supplementation ingestion on pathways promoting systematic inflammation with metabolic dysfunction-associated steatotic liver disease (MASLD) enabling a better understanding of the mechanisms of action. This includes effects on gut microbiome composition, body weight, lipid metabolism, and gut and liver physiology. |
| Trial Configuration | Single centre placebo controlled randomised dietary intervention study |
| Setting | Identification of participants from secondary care setting for research across research centres: Nottingham Digestive Diseases Centre, University of Nottingham; Nottingham Clinical Research Facility at Nottingham University Hospitals NHS Trust; and Sir Peter Mansfield Imaging centre |
| Sample size estimate | The sample size was calculated using Raosoft® calculator with Confidence level of 95% ± 5% and response distribution of (50%) to know the minimum sample size. The total sample size came out to be 14 per arm. The sample size of this study is equal to 15 per arm to obtain the statistical power for the study.  This calculation was based on a previous pectin pilot study which found significant differences in 4 inflammatory markers with n=14 healthy adult volunteers. |
| Number of participants | 45 |
| Eligibility criteria | **Inclusion criteria for the main study:**  -Patients with clinical diagnosis of MASLD (formerly termed non-alcoholic fatty liver disease (NAFLD)), having assessment suggesting that liver fat > 5% (e.g. histological evidence or/ and Transient Elastography using Controlled Attenuation Parameter (CAP)- FibroScan™ in the past month and/or liver imaging (such as ultrasound, computerized tomography (CT) or magnetic resonance imaging (MRI)).  -Participants willing and able to give informed consent for participation in the study.  -Participants aged ≥18 years who have a body mass index (BMI) between 18.5 and 39.9 kg/m^2^ and stable weight (weight gain or loss ≤ 3kg) for the past 3 months.  -For diabetic participants: controlled blood glucose levels Haemoglobin A1C (HbA1c) <7.0% (<53 mmol/mol) [1].  -Able to undergo CAP-FibroScan™.  **Inclusion criteria for healthy participants who will have MRI scans:**  -Participants willing and able to give informed consent for participation in the study.  -Participants aged ≥18 years.  - participants with CAP<250 kpa<8kP by a FibroScan™ within the past 6 months.  **Exclusion criteria for the main study:**  -Have allergy toward soya, milk or chocolate.  -Have allergy toward pectin.  -Participants on vegan diet.  -Have eating disorders or difficulties or gastrointestinal conditions e.g. malabsorptive conditions such as coeliac, Irritable Bowel Syndrome (IBS) or Inflammatory Bowel Disease (IBD) or gastroparesis.  -Have chronic malnutrition condition.  -History of major surgery which potentially limits participation or completion of the study.  -History of previous intestinal surgery known to affect food intake  or digestive function, including bariatric surgery.  -Use of antibiotics, antifungal medications, probiotics or prebiotics 90 days before the start of the study.  -Are taking the following medications: immunosuppressants, amiodarone and/or perhexiline.  -Are currently following or anticipated to commence a specialised commercially available weight loss diet and/or program or concomitant use of any weight loss medication or herbal weight loss products.  -History of side effects towards probiotics or prebiotics.  -History or current psychiatric illness.  -History or current neurological condition (e.g. epilepsy).  -Participants with other liver abnormalities.  -Evidence of monogenic metabolism diseases such as Lysosomal acid lipase deficiency (LALD), Wilson disease, Hypobetalipoproteinemia, or inborn errors of metabolism.  -Have had a weight change exceeding 3 kg within 3 months.  -Uncontrolled diabetes, active malignancy, or chronic infections.  -Having symptoms of active infection.  - Excessive alcohol intake defined as self-reported intakes greater than 21 units per week in men, and 14 units per week in women.  -Participants who are pregnant, breast feeding or actively planning pregnancy will be excluded from the study.  -Participation in any other trial in the last 3 months.  **Exclusion criteria for healthy volunteers MRI scans and patients optional MRI scans:**  -Contraindications for MRI scanning: having pacemakers, defibrillators, neurostimulators, prohibited medical implants, and foreign bodies (e.g. bullets, shrapnel, metal slivers), history of metallic foreign body in eye(s) and penetrating eye injury that could present a risk during an MRI scan.  -Difficulty breathing or inability to lie flat, as well as conditions that could worsen under stress (such as anxiety or panic disorders, claustrophobia, uncontrolled hypertension, or seizure disorders) severe enough to prevent undergoing an MRI.  -Contraindications to Hyoscine butylbromide (Buscopan®): Previous adverse drug reaction (allergic, hypersensitivity or other), Angle-closure glaucoma, tachycardia, ischaemic heart disease, myasthenia gravis, prostatic enlargement with urinary retention requiring catheterisation, mechanical stenosis in the gastrointestinal tract, paralytic or obstructive ileus, hypotension, cardiac disease, recent heart attack or any arrhythmias, thyrotoxicosis, gastro-oesophageal reflux disease, hiatus hernia, ulcerative colitis. |
| Description of interventions | Arm A: Pectin fibre (15g/day) combined with 15g whey protein supplement and 5g cocoa powder reconstituted as a shake Arm B (control/placebo): Whey protein supplement (30g/day) and 5g cocoa powder reconstituted as a shake.  To validate MRI scans as a tool to assess intestinal wall thickness to indicate gut permeability on MASLD patients, our team will scan 15 healthy volunteers twice, at baseline and after 6 weeks, and then compare their results with MASLD participant results at baseline and after 6 weeks. |
| Duration of study | Duration of the study:  24 months.  Duration per participant:  Each participant will participate for 6 weeks. |
| Randomisation and blinding | The study will be a parallel design, placebo-controlled, randomised dietary intervention study in which each participant will be randomly assigned to either pectin supplementation or control dietary supplement using online software (sealedenvelope.com). |
| Outcome measures | Primary outcomes:  Quantification of blood markers associated with physiological processes and pathways contributing to systemic inflammation pathway (TNFα, IL-6, IL-10, IFNᵞ, C-Reactive Protein, Zonulin (Haptoglobulin), IL-1β).  Secondary outcomes:   1. Assessment of changes in anthropometric measures 2. Assessment of changes in general metabolic indicators, such as fasting blood glucose and other blood-based markers relevant to MASLD (e.g., CK18-M30, CK18-M65, PROC3, Enhanced Liver Fibrosis (ELF), NIS2+™, YKL-40, microRNA miR-34a-5p, liver-associated enzymes such as Alanine Aminotransferase (ALT), Aspartate Aminotransferase (AST), gamma-glutamyl transferase (GGT), Alkaline Phosphatase (ALP)), bilirubin levels, lipid profiles, and platelet counts. 3. Exploration of modifications in non-invasive physiological assessments linked to liver characteristics, such as fat content and stiffness through controlled attenuation parameter (CAP) and transient elastography. 4. Observation of alteration in fat in liver and other surrounding abdominal organs through Dixon MRI sequence in patients who will agree to have 2 MRI scans. 5. Validation of MRI measures (T2*) as a tool to measure gut permeability among MASLD patients and investigation of changes in gut permeability in participants undergoing two MRI scans. 6. Investigation the presence of gene variants such as MUC2, encoding Mucin protein, that are associated with gut permeability. |
| Statistical methods | Data input, cleaning and analysis will be conducted using a statistical software such as PRISM or SPSS (IBM Corp, Armonk, New York) or R software (R Foundation for Statistical Computing, Vienna, Austria). Descriptive statistics will be reported as means ± standard deviations (SD) or medians with interquartile ranges (IQR) for continuous variables, and as frequencies and percentages for categorical variables. Prior to analysis, data will be checked for normality using the Shapiro-Wilk test. Standard parametric and non-parametric tests (where assumptions of normality are violated) will be used to assess significance of changes compared to baseline on both pectin arm and placebo arm. Mixed-effects linear regression models will be used to assess the intervention effect of pectin compared to placebo, adjusting for baseline values and relevant covariates such as age, sex, ethnicity and BMI. The interaction between time (baseline and follow-up) and group (pectin vs placebo) will be examined to determine if the changes over time differ between the two groups. P-value < 0.05 will be considered statistically significant for all analyses. |

# ABBREVIATIONS

AASLD American Association for the Study of Liver Disease

AE Adverse Event

ALP Alkaline Phosphatase

ALT Alanine Aminotransferase

AST Aspartate Aminotransferase

CAP Controlled attenuation parameter

CI Chief Investigator overall

CRF Case Report Form

CT Computerized tomography

DILI Drug-Induced Liver Injury

DMC Data Monitoring Committee

ELF Enhanced Liver Fibrosis

GABA gamma-aminobutyric acid

GCP Good Clinical Practice

GGT gamma-glutamyl transferase

GSRS Gastrointestinal Symptom Rating Scale

HADS Hospital Anxiety and Depression Scale

HBA1C Haemoglobin A1C

HCV Hepatitis C Virus

HM High methoxyl

HPA hypothalamic-pituitary-adrenal

IBD Inflammatory bowel disease

IBS Irritable bowel syndrome

ICF Informed Consent Form

IQR Interquartile ranges

LALD Lysosomal Acid Lipase Deficiency (LALD)

LM Low methoxyl

MASH Metabolic Dysfunction-associated Steatohepatitis

MASLD Metabolic dysfunction-associated steatotic liver disease

MRI Magnetic resonance imaging

NAFLD Non-alcoholic fatty liver disease

NASH Non-alcohol related steatohepatitis

NHS National Health Service

NICE National Institute for Health and Care Excellence

PI Principal Investigator at a local centre

PIS Participant Information Sheet

QOL Quality of life

REC Research Ethics Committee

R&D Research and Development department

SAE Serious Adverse Event

SD Standard deviation

SLD steatotic liver disease

SPMIC Sir Peter Mansfield Imaging Centre

VTCE Vibration-controlled transient elastography

**TABLE OF CONTENTS**

[SYNOPSIS 4](#_Toc189215193)

[ABBREVIATIONS 8](#_Toc189215194)

[TRIAL / STUDY BACKGROUND INFORMATION AND RATIONALE 12](#_Toc189215195)

[TRIAL / STUDY OBJECTIVES AND PURPOSE 17](#_Toc189215196)

[PURPOSE 17](#_Toc189215197)

[PRIMARY OBJECTIVE 17](#_Toc189215198)

[SECONDARY OBJECTIVES 17](#_Toc189215199)

[1. DETAILS OF PRODUCT(S) 17](#_Toc189215200)

[**Description** 17](#_Toc189215201)

[**Intervention Arm** 17](#_Toc189215202)

[**Manufacture** 19](#_Toc189215203)

[**Packaging and labelling** 19](#_Toc189215204)

[**Storage, dispensing and return** 20](#_Toc189215205)

[**Known Side Effects** 20](#_Toc189215206)

[TRIAL / STUDY DESIGN 21](#_Toc189215207)

[TRIAL / STUDY CONFIGURATION 21](#_Toc189215208)

[Primary endpoint 21](#_Toc189215209)

[Secondary endpoint 21](#_Toc189215210)

[Stopping rules and discontinuation 21](#_Toc189215211)

[RANDOMIZATION AND BLINDING 22](#_Toc189215212)

[Maintenance of randomisation codes and procedures for breaking code 22](#_Toc189215213)

[TRIAL/STUDY MANAGEMENT 22](#_Toc189215214)

[DURATION OF THE TRIAL / STUDY AND PARTICIPANT INVOLVEMENT 22](#_Toc189215215)

[End of the Trial 22](#_Toc189215216)

[SELECTION AND WITHDRAWAL OF PARTICIPANTS 23](#_Toc189215217)

[Recruitment of patients for the study 23](#_Toc189215218)

[Recruitment of healthy volunteers 23](#_Toc189215219)

[Eligibility criteria 24](#_Toc189215220)

[Inclusion criteria for the main study 24](#_Toc189215221)

[Exclusion criteria for the main study 24](#_Toc189215222)

[Expected duration of participant participation 25](#_Toc189215223)

[Study participants will be participating in the study for 6 weeks, attending two 2 hours study visits. Participants who will agree to have MRI scans, including healthy volunteers, will need to attend two 4 hours MRI visits. 25](#_Toc189215224)

[Removal of participants from therapy or assessments/Participant Withdrawal 25](#_Toc189215225)

[Informed consent 25](#_Toc189215226)

[TRIAL / STUDY INTERVENTION AND REGIMEN 26](#_Toc189215227)

[Compliance 30](#_Toc189215228)

[Criteria for terminating trial 30](#_Toc189215229)

[TRANSPORT AND STORAGE OF THE TISSUES 30](#_Toc189215230)

[LABORATORY ANALYSES 31](#_Toc189215231)

[STATISTICS 31](#_Toc189215232)

[Methods 31](#_Toc189215233)

[Sample size and justification 31](#_Toc189215234)

[Assessment of efficacy 31](#_Toc189215235)

[Primary endpoint 32](#_Toc189215236)

[Secondary endpoint 32](#_Toc189215237)

[Assessment of safety 32](#_Toc189215238)

[Procedures for missing, unused and spurious data 33](#_Toc189215239)

[Definition of populations analysed 33](#_Toc189215240)

[ADVERSE EVENTS 33](#_Toc189215241)

[Definitions 33](#_Toc189215242)

[Causality 34](#_Toc189215243)

[Reporting of adverse events 35](#_Toc189215244)

[Trial Intervention Related SAEs 35](#_Toc189215245)

[Participant removal from the study due to adverse events 35](#_Toc189215246)

[ETHICAL AND REGULATORY ASPECTS 35](#_Toc189215247)

[ETHICS COMMITTEE AND REGULATORY APPROVALS 35](#_Toc189215248)

[INFORMED CONSENT AND PARTICIPANT INFORMATION 36](#_Toc189215249)

[RECORDS 36](#_Toc189215250)

[Case Report Forms 36](#_Toc189215251)

[Sample Labelling 37](#_Toc189215252)

[Source documents 37](#_Toc189215253)

[Direct access to source data / documents 37](#_Toc189215254)

[DATA PROTECTION 37](#_Toc189215255)

[QUALITY ASSURANCE & AUDIT 37](#_Toc189215256)

[INSURANCE AND INDEMNITY 38](#_Toc189215257)

[TRIAL CONDUCT 38](#_Toc189215258)

[TRIAL DATA 38](#_Toc189215259)

[RECORD RETENTION AND ARCHIVING 38](#_Toc189215260)

[DISCONTINUATION OF THE TRIAL BY THE SPONSOR 38](#_Toc189215261)

[STATEMENT OF CONFIDENTIALITY 39](#_Toc189215262)

[PUBLICATION AND DISSEMINATION POLICY 39](#_Toc189215263)

[USER AND PUBLIC INVOLVEMENT 39](#_Toc189215264)

[STUDY FINANCES 40](#_Toc189215265)

[Funding source 40](#_Toc189215266)

[Participant stipends and payments 40](#_Toc189215267)

[SIGNATURE PAGES 40](#_Toc189215268)

[REFERENCES 41](#_Toc189215269)

#

# TRIAL / STUDY BACKGROUND INFORMATION AND RATIONALE

**Metabolic Dysfunction-Associated Steatotic Liver Disease (MASLD): Definition and Prevalence**

Metabolic dysfunction-associated steatotic liver disease (MASLD), formerly known as non-alcoholic fatty liver disease (NAFLD), is the main cause of chronic liver disease and affects more than 32% adults globally. Meta-analysis studies across the world showed that the predicted incidence of MASLD is 47 cases per 1000 people with higher incidence in obese and overweight males [2]. Moreover, in the UK, this disease is affecting 1 in 5 people and is the most common liver disorder. The number of new cases is increasing, and it is estimated that MASLD/NAFLD will become the main reason for liver transplantation within the coming years [3, 4].

NAFLD- is defined as an accumulation of fat within the liver cells (steatosis), where the presence of fat is more 5% in the hepatic cells, and in the absence of other liver disease causes or significant alcohol intake. Significant alcohol consumption is defined by American Association for the Study of Liver Disease (AASLD) as recent or current alcohol use of >21 drinks/week in men and >14 drinks/week in women [5].

NAFLD includes a range of liver-related abnormalities. While most patients have simple steatosis, a smaller group of these patients can develop non-alcoholic steatohepatitis (NASH), a more progressive form of liver disease that is defined as the presence of hepatic steatosis with evidence for liver cell injury (apotosis) and characterised by inflammation and fibrosis. In later stages, NASH can progress to ongoing hepatic fibrosis, then to cirrhosis and can lead to life-threatening conditions including liver cancer and liver failure [6, 7].

MASLD as a terminology has been recently introduced by AASLD in June 2023 to replace NAFLD to accurately capture the cause of liver steatosis. Based on the new guidelines, patients will be diagnosed with MASLD if they have liver steatosis and have at least one of five cardiometabolic risk factors: obesity (BMI ≥25kg/ m2 (23 in Asians) or waist circumference >94cm in male, >80cm in female or ethnicity adjusted), insulin resistance (fasting serum glucose ≥ 5.6 mmol/l (100 mg/dl) or 2-hour post-load glucose levels ≥ 7.8 mmol/l (≥ 140 mg/dl) or HbA1c ≥ 5.7% (39 mmol/l) or type 2 diabetes or treatment for type 2 diabetes), hypertension (blood pressure ≥ 130/85 mmHg or specific antihypertensive drug treatment) and dyslipidaemia (plasma triglycerides ≥ 1.70 mmol/l (150 mg/dl) or lipid-lowering treatment or plasma HDL-cholesterol ≤ 1.0 mmol/l (40 mg/dl) (male) and ≤ 1.3 mmol/l (50 mg/dl) (female) or lipid-lowering treatment. NASH has been also replaced by Metabolic dysfunction-associated steatohepatitis (MASH) to indicate that steatohepatitis is related to metabolic dysfunction. Moreover, an overarching term of steatotic liver disease (SLD) was introduced by AASLD to cover the different causes of liver steatosis and a new category, separate from MASLD, called MetALD was introduced to classify individuals with MASLD who consume higher weekly amounts of alcohol—specifically over 140 grams (14 drinks) for females and 210 grams (21 drinks) for males [8, 9]. These subtypes are shown in Figure 1


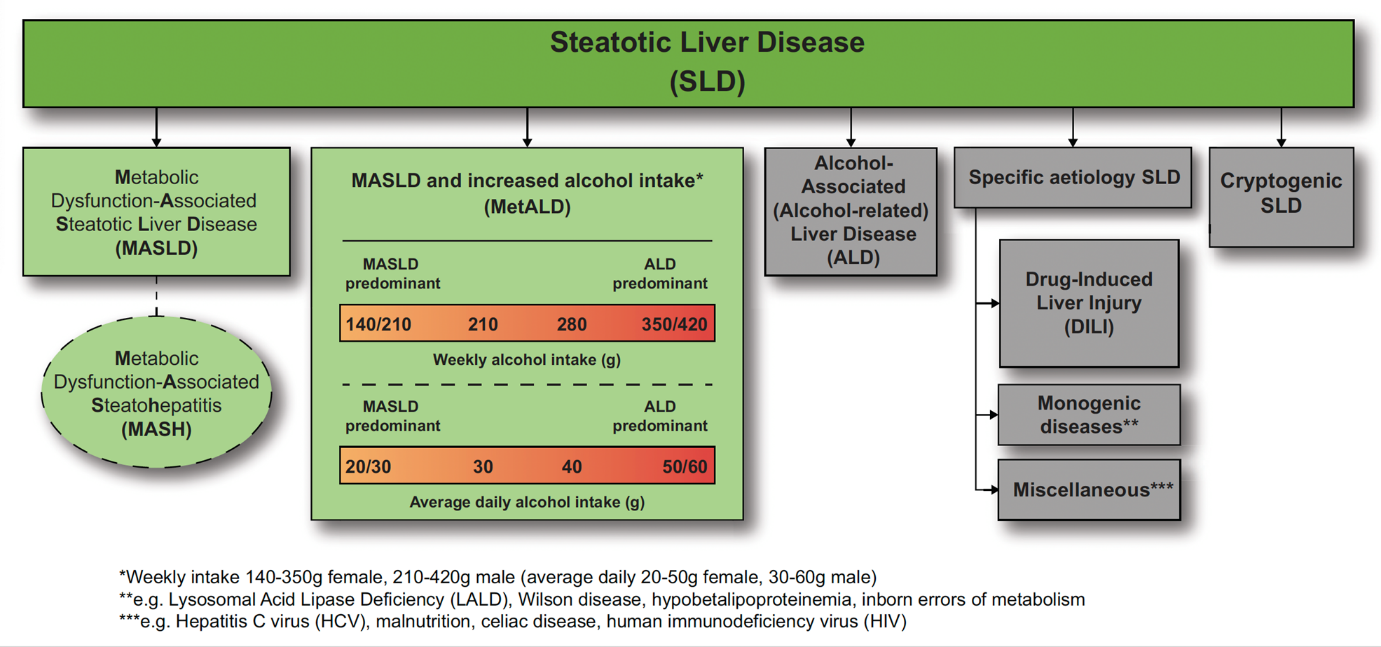


Figure 1: Reproduced from [9]

**The Pathological Development of MASLD**

The pathogenesis of MASLD has been linked to the presence of one or more of the following factors: impaired lipid metabolism, insulin resistance, increased gut permeability, high-starch carbohydrates and high-fat diets, genetic factors, alteration in the inflammatory signalling and immune system pathways, endoplasmic reticulum stress and oxidative stress. All these factors play important role in the progression of MASLD to more severe stages of liver disease [10, 11].

*Gut permeability and MASLD*

The liver receives blood through both arterial and venous systems, with most of its blood flow coming from the intestines via the portal vein. This connection exposes the liver to potentially harmful substances from the gut, including translocated bacteria, lipopolysaccharides (LPS) and secreted cytokines. A primary function of the liver is to remove these substances from the bloodstream. Gut permeability is defined as weakened intestinal barrier. There are several proteins that are responsible for sealing the junctions between intestinal endothelial cells, playing a critical role in preventing harmful compounds from passing into the portal circulation. However, dysbiosis (disruption of the normal gut microbiota) can disrupt these tight junctions, leading to increased intestinal permeability and exposing both the gut lining and liver to inflammatory bacterial byproducts [12].

Growing research suggests that there is a link between gut health and the development of various liver conditions, such as MASLD and MASH. This was based on findings which indicated that individuals with MASLD exhibit higher intestinal permeability, endotoxemia (increased of plasma level of LPS) and increased bacterial overgrowth in the small intestine compared to those without the condition [13]. Additionally, liver disease has been linked to alterations in gut bacterial composition, and modifying the gut flora has been shown to influence liver damage [6].

The gut microbiota provides additional energy for the body by breaking down polysaccharides into short-chain fatty acids (SCFAs) such as acetic, propionic and butyric acid through Bacteroides. SCFAs serve as energy sources for the liver, where they play key roles in hepatic lipogenesis (fat production) and gluconeogenesis (the generation of glucose), respectively. Acetate, in particular, can be utilized as a precursor for cholesterol or fatty acid synthesis. SCFAs contribute approximately 30% of the liver's energy needs. The types and concentrations of SCFAs produced in the gut are affected by carbohydrate intake and dysbiosis, therefore alteration in gut microbiota can affect SCFAs and by that affect liver lipogenesis and gluconeogenesis [12]. Moreover, SCFAs interact with receptors such as G-protein-coupled receptors (e.g., GPR41 and GPR43) found on immune cells, influencing immune and inflammatory responses. By signalling through these receptors, SCFAs may either exacerbate or reduce inflammatory pathways, depending on their concentrations and balance within the gut lining. Butyrate, for example, may help reduce inflammation by influencing regulatory T cells in the gut lining. A human study found that faecal transplants from lean donors to patients with obesity led to significant metabolic improvements, which were associated with a notable increase in the abundance of Roseburia intestinalis, a key butyrate-producing bacterium. The overall impact of SCFAs on intestinal inflammation and permeability likely depends on the specific balance of SCFAs generated in the gut [12, 14].

Certain gut bacteria have been linked to the development of MASLD; for example, studies on obese mice have shown a 50% reduction in Bacteroides and an increase in Firmicutes compared to lean mice. Furthermore, germ-free mice exhibit a significant rise in body fat when colonized with microbiota from obese donors. On the other hand, a newly identified bacterium, Akkermansia muciniphila, is associated with a leaner body type in both humans and animal studies; high-fat diet mice treated with Akkermansia show less inflammation in fat tissue and improved glucose tolerance. The gut flora also produces enzymes that break down dietary choline—a component crucial for cell membrane integrity and lipid transport in the liver—into methylamines, which are harmful compounds linked to inflammation and liver damage. Dysregulated microbiota may lead to triglyceride buildup and exacerbate MASLD by decreasing choline availability and increasing methylamine levels [6].

There are several genes affecting the building of the epithelial barrier and the luminal microbiota and therefore their variants might increase gut permeability. One of these genes is MUC2. MUC2 is responsible for producing mucin, a key component of the mucus layer that protects the gut lining. MUC2 directly contributes to forming a physical barrier that prevents bacteria and toxins from reaching the epithelial cells of the gut. A study that was done on MUC2 knockout mice proved that absence of MUC2 increased gut permeability. Therefore, downregulation or mutation of MUC2 can increase gut permeability by reducing mucin levels [15, 16].

Gut permeability is widely measured through tracking the urinary excretion of certain sugars or sugar alcohols (e.g., lactulose, mannitol, rhamnose, sucralose), which are absorbed in the gut but minimally metabolised. The lactulose-to-mannitol excretion ratio (LMR) is the most widely used and validated marker of permeability and has even been adopted as an endpoint in clinical trials. Recent research, that has been conducted at the University of Nottingham, has validated MR measures of small bowel wall T2* as a non-invasive novel technique to investigate gut permeability in healthy volunteer [17].

**Non-invasive tests for diagnosing MASLD**

Based on National Institute for Health and Care Excellence (NICE) guidelines, liver biopsy is still the gold standard to diagnose patients with MASLD, however, it is not feasible to use it to screen all people with risk factors (central obesity (excessive abdominal fat), insulin resistance or type 2 diabetes, hypertension, and dyslipidaemia) because it is invasive, has risk of complications, and is expensive. Other non-invasive diagnostic tools include clinical, biochemical and radiographic tests such as Fibroscan™ controlled attenuation parameter (CAP). MRI can also be used to determine liver fat content [18, 19].

**Project Rationale**

Pectin is a complex soluble heteropolysaccharides that is found in the primary cell walls of higher plants and commonly used as a food additive. Pectin is classified based on the esterification degree to either high-methoxy (HM) pectin where the esterification degree is higher than 50% or Low-methoxy (LM) pectin where the esterification degree is lower than 50%. HM pectin is usually difficult to be digested or absorbed by human bodies. In contrast, LM pectin is reported to modify the gut microbiome and its metabolites, and on systematic inflammation pathway by reducing pro-inflammatory cytokines [20, 21].

*Research question*

How does dietary Low-methoxy (LM) pectin supplementation affect systematic inflammation pathways such as those mediated by gut microbiota composition and what are the impacts on general metabolic indicators in individuals with MASLD?

*Hypothesis*

LM pectin could potentially modulate gut microbiota and improve gut permeability; and could interact with Toll-like receptors (TLRs) and inhibit of their activity [15, 16]. These mechanisms are explained below.

1. Pectin effects on gut microbiota and gut permeability

- Short-Chain Fatty Acids (SCFAs) production

Pectin fermentation by gut bacteria produces a wide range of metabolites including SCFAs such as propionate and butyrate. SCFAs can play important role in lipid and glucose homeostasis by entering the peripheral circulation through portal vein and then acting on the liver and peripheral tissues. Moreover, inside the intestine, short-chain fatty acids can enhance the secretion of peptide YY (PYY) and Glucagon-like peptide 1 (GLP-1) by enteroendocrine cells and both peptides are responsible for slowing down the intestinal transit and supressing food intake. Both in vivo and in vitro studies have showed that pectin can increase SCFAs. Moreover, research has indicated that pectin can strengthen gut barrier function and reduce permeability by increasing the amount of SCFAs that can promote the production of mucus in the intestines of rodents and improve the building of tight junction. Studies showed that LM pectin produced higher amount of SCFAs compared to HM pectin [22-24].

- Modulate microbiota

The effect of pectin on gut microbiota has been studied widely through in vivo and in vitro models. Studies have shown that this effect is variable depending on the degree of esterification, chemical structure and the molecular weight of pectin. Prior studies involving rats, pigs, and humans, indicated that pectin can stimulate the growth of beneficial bacteria such as Bacteroidetes and Firmicutes. Studies reported a reduction in Bacteroidetes within the gut of MASLD patients [22, 25].

- Strengthening tight junctions

LM pectin helps reinforce the structure and function of tight junctions (protein complexes connecting epithelial cells in the gut lining). Tight junctions regulate the selective permeability of the gut, allowing nutrients and water to pass while blocking harmful substances. Studies suggest that LM pectin increases the expression of proteins involved in tight junction integrity, such as occludin. This reinforcement reduces the likelihood of molecules leaking through the intestinal barrier [26].

1. The anti-inflammatory effects of pectin

- Binds directly to Toll-Like Receptors (TLRs) and inhabits their inflammatory activity

Research using human and animal models has demonstrated that pectin has a potential anti-inflammatory effect through interacting with Toll-Like Receptors (TLRs). TLRs play crucial role in the progression of liver inflammation and steatosis. TLR2 and TLR4 are the most common TLRs associated with metabolic inflammation. TLR2 up-regulation was reported in many NAFLD animal models as well as led to development of NASH in in the liver of E3 rats. In addition, anti-TLR2 antibody reduces hepatic injury, inflammation, fibrosis and steatosis in rats with obesity-related metabolic disorder via regulation of MAPK and NF-κB pathways. Using pectin, as a promising TLR2 inhibitor [27, 28].

Pectin can directly bind to TLRs and inhibit their activity based on its degree of esterification; the higher the degree of methyl-esterification, the lower the binding to TLRs. LM pectin was able to strongly inhibit TLR2-1 induced IL-6 secretion in human macrophages [29]. In in vitro studies that included mouse macrophages and human dendritic cells, LM pectin showed an anti-inflammatory response by directly binding to TLR2–TLR1 receptors. This effect was independent of the effects due to SCFAs produced by the gut microbiota [30].

A recent study by our group at University of Nottingham showed that 4 weeks consumption of a daily supplement containing 15-20g of LM pectin significantly reduced pro-inflammatory blood markers such as TNFα (p<0.003), increased anti-inflammatory blood marker IL-10 (p=0.08), decreased TLR-2 concentration in the blood (p<0.027) and improved gut permeability with no recorded side effects in healthy volunteers [31].

- Short-Chain Fatty Acids (SCFAs) production

As mentioned above, butyrate, one of SCFAs that is produced after LM pectin fermentation in the gut, may help reduce inflammation by influencing regulatory T cells in the gut lining that Can lead to increase the level anti-inflammatory cytokines like IL-10 [14]. This might reduce the systematic inflammation.

The diagram below summarises the effects of LM pectin on gut microbiota composition and systematic inflammation pathways in individuals with MASLD:


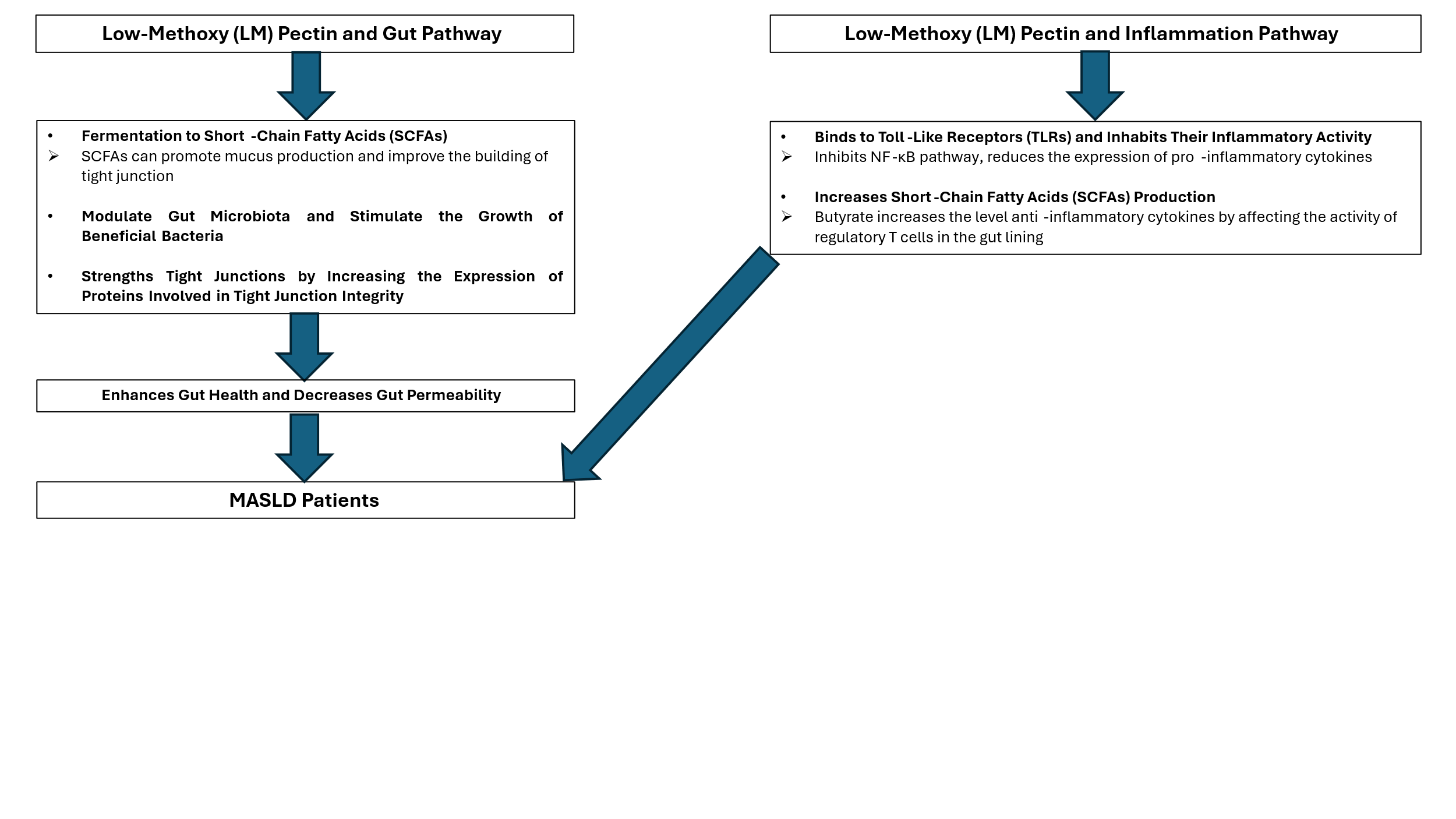


*Study aims*

Therefore, the current research aims to study the effects of LM pectin, the dietary supplementation intervention, on pathways contributing to systematic inflammation in patients with MASLD.

# TRIAL / STUDY OBJECTIVES AND PURPOSE

## PURPOSE

This research project will explore the effects of pectin supplementation on systemic inflammation and gut microbiome composition and the consequences of these effects on general metabolic indicators, in individuals diagnosed with MASLD.

## PRIMARY OBJECTIVE

This research aims to study the effects of daily ingestion of LM pectin on inflammation pathways by measuring the blood inflammatory markers associated with physiological processes (TNFα, IL-6, IL-10, IFNᵞ, C - reactive protein, Zonulin (Haptoglobulin), IL-1β).

## SECONDARY OBJECTIVES

1. Assessment of changes in anthropometric measures.
2. Assessment of changes in general metabolic indicators, such as fasting blood glucose and other blood-based markers relevant to MASLD (e.g., CK18-M30, CK18-M65, PROC3, Enhanced Liver Fibrosis (ELF), NIS2+™, YKL-40, microRNA miR-34a-5p, liver-associated enzymes such as Alanine Aminotransferase (ALT), Aspartate Aminotransferase (AST), gamma-glutamyl transferase (GGT), Alkaline Phosphatase (ALP)), bilirubin levels, lipid profiles, and platelet counts.
3. Exploration of changes in gut microbiome composition.
4. Exploration of modifications in non-invasive physiological assessments linked to liver characteristics, such as fat content and stiffness through controlled attenuation parameter (CAP) and transient elastography.
5. Observation of alteration in fat in liver and other surrounding abdominal organs through Dixon MRI sequence in patients who will agree to have 2 MRI scans.
6. Validation of MRI measures (T2*) as a tool to measure gut permeability among MASLD patients and investigation of changes in gut permeability in participants undergoing two MRI scans.
7. Investigation the presence of gene variants such as MUC2, encoding Mucin protein, that are associated with gut permeability.
8. **DETAILS OF PRODUCT(S)**

**Description**

**Intervention Arm**

**Pectin (active substance)**

LM citrus pectin (CAS 9000-69-5) food grade pectin containing >65% galacturonic acid and ≤50 mg/kg Sulphur Dioxide

Certified as Kosher

Source: Polysaccharide found in cell walls of all plants

Appearance: White to light brown powder

Solubility: Soluble in Water (Very Slightly)

Refractive Index: 1.543

Stability: Stable under normal shipping and handling conditions

Usual Daily Dose: 5-20g

Storage: Store at room temperature

**Cocoa Powder (flavour substance)**

Nesquik®

Contains: Sugar, Fat-Reduced Cocoa Powder (23%), Emulsifier (Soya Lecithin), Salt, Vitamins (C, D), Natural Flavouring, Cinnamon.

Appearance: brown powder

Solubility: Soluble in Water

Stability: Stable under normal shipping and handling conditions

Usual Daily Dose: 4.5-9g

Storage: Store at room temperature

**Protein Powder**

Whey protein

Contains: Whey protein isolate (milk) (100%), Emulsifier (Soya Lecithin, Sunflower Lecithin)

Per 100g of whey protein: Fat 0.9g, of which saturates 0.4g, Carbohydrate 2.8g, of which sugars 1.7g, Salt 0.68g, 86g Protein

Appearance: White powder

Solubility: Soluble in Water

Stability: Stable under normal shipping and handling conditions

Usual Daily Dose: 20-30g

Storage: Store at room temperature in cool and dry place away from sunlight.

**Placebo Arm**

**Cocoa Powder (flavour substance)**

Nesquik®

Contains: Sugar, Fat-Reduced Cocoa Powder (23%), Emulsifier (Soya Lecithin), Salt, Vitamins (C, D), Natural Flavouring, Cinnamon.

Appearance: brown powder

Solubility: Soluble in Water

Stability: Stable under normal shipping and handling conditions

Usual Daily Dose: 4.5-9g

Storage: Store at room temperature

**Protein Powder**

Whey protein

Contains: Whey protein isolate (milk) (100%), Emulsifier (Soya Lecithin, Sunflower Lecithin)

Per 100g of whey protein: Fat 0.9g, of which saturates 0.4g, Carbohydrate 2.8g, of which sugars 1.7g, Salt 0.68g, 86g Protein

Appearance: White powder

Solubility: Soluble in Water

Stability: Stable under normal shipping and handling conditions

Usual Daily Dose: 20-30g

Storage: Store at room temperature in cool and dry place away from sunlight

**MRI Scans**

**Buscopan® (will be used during MRI scans as antispasmodics)**

Active ingredients: Hyoscine butylbromide 20 mg per 1 ml

Licensing authority: Medicines - MHRA/EMA

Size: 10

Unit: Ampoule

Usual Dose: 20 mg, then 20 mg after 30 minutes if required, dose may be repeated more frequently; maximum 100 mg per day

Storage: Store in a cool, dry place, away from direct heat and light

**Mannitol 2.5% / Locust bean gum 0.2% oral solution ( will be used during MRI scans as oral contrast)**

Mannitol 2.5% / Locust bean gum 0.2% oral solution

Licensing authority: None

Pack size: 1000ml

Unit: Oral Solution

**Manufacture**

**Pectin**

BOC SCIENCES INC

45-16 Ramsey Road

Shirley

New York

11967 Massachussetts

United States

**Cocoa Powder (Nesquik®)**

NESTLE UK LTD

Haxby Road

York, England

YO31 8TA

United Kingdom

**Protein Powder**

Myprotein a THG company

BRGCS AA+ Certified

THG Nutrition Limited

7-9 Sunbank Lane

Altrincham

WA15 0AF

United Kingdom

**Buscopan®**

Sanofi

410 Thames Valley Park Drive

Reading

Berkshire

RG6 1PT

**Mannitol**

Life Lab Supplements

Unit D Evolve At Vision Park Bell Way,

Burnley

Lancashire

BB12 0BS

**Locust bean gum**

Special Ingredients Ltd

Foxwood Industrial Park

Chesterfield

S41 9RN

**Packaging and labelling**

The study team will repackage the powders into zip lock bags labelled only as the study arm A or B based on a local SOP that was written by the study team. The repackage process will take place at a clean room at School of Medicine, University of Nottingham, E-Floor by a food hygiene trained researcher. The researcher will use electronic food scale to weigh the powders. All prepared zip bags will be kept in a dry secure location. This will be in a locked cabinet in E Floor, Nottingham Digestive Diseases, Queens Medical Centre, Nottingham, NG7 2UH and only accessible by designated study researchers. Participants will be provided the supplements mixed with the flavour in clear plastic food safe zip lock bags. Each bag will contain either 15g of pectin with 5g of cocoa powder and 15g of whey protein powder or 30g of whey protein powder with 5g of cocoa powder.

Participants will be advised to pour 250 ml of water into a shaker bottle, then empty the whole powder inside the bag and shake the bottle until they have consistent shake. They will be advised to add water up to 50 ml if required to enhance solubility. Participants will be advised to consume one bag per day, each participant will be given 42 zip bags to be consumed within 42 days. A brochure with easy instructions will be given to each participant.

Participants will be advised to take the intervention in the morning. If they forget to take it in the morning, they will be advised to take it once they remember. Moreover, participants will be advised to avoid missing doses or overdoses as much as they can and to report missing doses or overdoses incident to the study team if that will happen. They will be given a tracker to track their daily study supplement consumption.

The study team will use pectin that is provided from BOC SCIENCES INC, United States similar to the pectin that was used on the healthy volunteer study that was previously conducted at the University of Nottingham [31].

Mannitol 2.5% / Locust bean gum 0.2% will be used as an oral contrast during u scans. It will be an oral solution that will be prepared by the MRI research team on the MRI visit day. Each drink will contain 25g of Mannitol and 2g of Locust bean gum that will be dissolved in 1000 ml of water.

Buscopan® intravenous injection will be used during MRI scans as antispasmodics. It will be prescribed, dispatched then administered by a medical doctor during MRI visit through cannula. The medical doctor will be a member of the University of Nottingham research team.

**Storage, dispensing and return**

Pectin, whey protein, and cocoa powder are safe to be stored at room temperature. They will be kept in sealed containers in a dry location. This will be in a locked cabinet in E Floor, Nottingham Digestive Diseases, Queens Medical Centre, Nottingham, NG7 2UH and only accessible by designated study researchers. Study Participants will be advised to bring back any unused zip bags and give them to the study team during follow up visits. Unused stock will be disposed as food waste into landfill waste.

Mannitol and Locust bean gum are safe to be stored at room temperature. They will be kept in sealed containers in a dry location. This will be in a locked cabinet in Sir Peter Mansfield Imaging Centre (SPMIC) and only accessible by the MRI research team. The contrast will be prepared and dispensed by the MRI research team.

Buscopan® will be stored at Sir Peter Mansfield Imaging Centre (SPMIC) and only accessible by the MRI research team. A qualified medical doctor, who will be a member of the research team too, will prescribe and dispense Buscopan® during the MRI visit.

**Known Side Effects**

As this is a dietary intervention trial using widely available foodstuffs consumed as advised by manufacturers, no serious adverse events/side-effects or ethical issues are expected. Participants will be given the details of the trial supplements - those with food allergies or sensitivities to components of the foods/components will not be recruited. Increasing fibre intake may result in bloating amongst those who have a low habitual intake of dietary fibre. However, such effects are mild and not considered as a safety concern. Presence of inflammatory bowel disease, irritable bowel syndrome, and gastrointestinal resection surgery are exclusion criteria for the study to avoid exacerbating these conditions. Pregnant and breastfeeding women will be excluded from the study because there is limited data regarding the safety of pectin and whey protein consumption during pregnancy and breastfeeding.

The common side effect from administration of the Buscopan® (that will be used during MRI scans as antispasmodics) are very short lived (blurred vision and raised heart rate) and participants will be advised to stay at the test centre until their vision returns to normal (this normally occurs within 30 minutes of the injection). Rare side effects from administration of the Buscopan® injection include skin reactions, tachycardia, dry mouth, dyshidrosis, and in extremely rare cases angle-closure glaucoma. This will be monitored throughout the study visit at Sir Peter Mansfield Imaging Centre (SPMIC) by a registered medical doctor who will be a member of the research team and who will administer Buscopan® and monitor participants.

Mannitol 2.5% / Locust bean gum 0.2% will be used during MRI scans as oral contrast. Mannitol increases MRI contrast between the lumen and the bowel wall, and the addition of locust bean gum serves to counteract some of the recognised side effects associated with Mannitol ingestion, which can include flatulence and alteration of gastrointestinal microbiota leading to bowel spasms and diarrhoea.

# TRIAL / STUDY DESIGN

## TRIAL / STUDY CONFIGURATION

This a single centre placebo controlled randomised dietary intervention study in which participants will be grouped into the intervention (A) or placebo arm (B). Randomisation will be controlled for equal distribution of key characteristics (e.g. sex, ethnicity) that may confound between group comparisons and will be assessed by analysis of baseline data.

### Primary endpoint

Levels of serum inflammatory biomarker such as; IL-6, IL-10, C-RP, Zonulin (Haptoglobulin), IL-1β, TNFα, IFNᵧ.

### Secondary endpoint

- Change in BMI
- Change in general metabolic indicators such as fasting blood glucose
- Changes in blood-based markers relevant to MASLD (e.g., CK18-M30, CK18-M65, PROC3, Enhanced Liver Fibrosis (ELF), NIS2+™, YKL-40, microRNA miR-34a-5p, liver-associated enzymes such as Alanine Aminotransferase (ALT), Aspartate Aminotransferase (AST), gamma-glutamyl transferase (GGT), Alkaline Phosphatase (ALP)), bilirubin levels, lipid profiles, and platelet counts.
- Gut microbiome: changes in composition
- Change in non-invasive physiological assessments linked to liver characteristics: FibroScan™ CAP value and Liver stiffness based on vibration-controlled transient elastography (VCTE)
- Change in liver fat and other surrounding abdominal organs fat through alterations in Dixon MRI sequence
- Alterations in T2* MRI measures of intestinal permeability

### Stopping rules and discontinuation

Since the current study is a dietary intervention, research team do not foresee any adverse events. However, if a participant develops a severe adverse reaction following consumption of the supplement (i.e. severe bloating amongst those who have a low habitual intake of dietary fibre), they will be asked to contact the study team immediately. Based on the severity of the event and at the discretion of the CI, the study team may advise the participant to continue or withdraw from the study.

Moreover, participants who will agree to have MRI scans, will be carefully monitored during the scan and if their medical or physiological status alter rapidly, then it may be necessary to stop scanning based on the radiologist advice.

Participants may withdraw from the study at any point. Significant non-compliance with the study schedule may lead to a participant being withdrawn from the study.

## RANDOMIZATION AND BLINDING

The study will be a parallel design, placebo controlled, randomised intervention study in which participants will be allocated to either the intervention or placebo arm. The randomisation will be done through an online software (sealedenvelope.com) to determine intervention A or B at enrolment.

One of research team will be un-blinded and will be responsible for preparing and labelling of the intervention/placebo as either A or B based on random selection using an online software (sealedenvelope.com). A or B – pectin will be assigned to the arm in the first envelope. This information will be securely documented in a new sealed enveloped stored in the chief investigators locked desk. All other members of the team will be blinded to the assignment of A or B as the intervention.

This will be unblinded once the study is complete after the sample analysis and data analysis is complete.

### Maintenance of randomisation codes and procedures for breaking code

The assignment of pectin as intervention A or B will be documented in a sealed envelope in the CI’s desk. This can be accessed and broken, if necessary, on direction of the CI in the event of any serious adverse event.

## TRIAL/STUDY MANAGEMENT

The CI has overall responsibility for the study and shall oversee all study management.

The data custodian will be the Chief Investigator.

The study will form part of PhD project undertaken by Noor Al-Tameemi registered at the University of Nottingham (UoN) whose role will include responsibility for recruiting and consenting patients and collecting data. The PhD student will be responsible for arranging and conducting study visits, analysing samples and analysing the data. The student will comply with University of Nottingham policies and regulations.

## DURATION OF THE TRIAL / STUDY AND PARTICIPANT INVOLVEMENT

Study Duration: 24 Months.

Recruitment: 22 months.

Follow up: 1.5 months.

The study is projected to have a total duration of 24 months from the start date.

Participant Duration:

6 weeks (+/- 5 days)

### End of the Trial

The end of the study will be the date of the last visit of the last participant or the completion of all follow-up monitoring, data collection, and required tissue sample analysis for the study participants, whichever is later..

## SELECTION AND WITHDRAWAL OF PARTICIPANTS

###

### Recruitment of patients for the study

The study will be set in secondary care at Nottingham University Hospitals NHS Trust through partnership with the University of Nottingham BRC.

Participants will be recruited through:

- Advertisements through posters, social media platforms and local websites:

Patient posters containing study information will be advertised in areas of the University and in the relevant clinical areas at Nottingham University Hospitals NHS Trust such as the Hepatology Department. Moreover, study will be advertised through University of Nottingham and NIHR Nottingham Biomedical Research Centre –local websites and social media platforms using the same patient posters. Study team will directly contact individuals who express interest in participating in the study.

- Approaching patients at hepatology clinics:

Participants will also be recruited from hepatology clinics. The initial approach will be from a member of the patient’s usual care team (which may include the investigator). Information about the trial will be on display in the relevant clinical areas.

The investigator or their nominee, e.g. from the research team or a member of the participant’s usual care team, will inform the participant of all aspects pertaining to participation in the study.

- Approaching participants with NAFLD/MASLD diagnosis who previously participated in research studies where Guru Aithal was the CI and who have consented to be contacted for future research.

Designated members of the study team will contact past research participants using contact details previously provided or obtained by the CI from patient records, to invite them to participate in this study.

It will be explained to the potential participant that entry into the trial is entirely voluntary and that their treatment and care will not be affected by their decision. It will also be explained that they can withdraw at any time, but attempts will be made to avoid this occurrence. In the event of their withdrawal, it will be explained that their data collected so far cannot be erased and we will seek consent to use the data in the final analyses where appropriate.

### Recruitment of healthy volunteers

A research team from the University of Nottingham has developed an MRI protocol to assess intestinal wall thickness to indicate gut permeability on healthy participants [17]. To validate this tool to be used on MASLD patients, our team will scan 15 healthy volunteers twice, at baseline and after 6 weeks, and then compare their results with MASLD patients’ results at baseline and after 6 weeks. Participants will be recruited through:

- Approaching healthy participants, with CAP<250 kpa<8kP by a FibroScan™ within the past 6 months, who previously participated in research studies where Guru Aithal was the CI and who have consented to be contacted for future research. Designated members of the study team will contact past research participants using contact details previously provided or obtained by the CI from patient records, to invite them to participate in this study.

### Eligibility criteria

### Inclusion criteria for the main study

- Patients with clinical diagnosis of MASLD, having assessment suggesting that liver fat > 5% (e.g. histological evidence or/ and Transient Elastography using CAP- FibroScan™ in the past month and/or liver imaging (such as CT or magnetic resonance imaging MRI)).
- Participants willing and able to give informed consent for participation in the study.
- Participants aged ≥18 years who have a body mass index (BMI) between 18.5 and 39.9 kg/m^2^ and stable weight (weight gain or loss ≤ 3kg) for the past 3 months.
- For diabetic participants: controlled blood glucose levels Haemoglobin A1C (HbA1c) <7.0% (<53 mmol/mol) [1].
- Able to undergo CAP-FibroScan™.

**Inclusion criteria for healthy participants who will have MRI scans:**

- Participants willing and able to give informed consent for participation in the study.
- Participants aged ≥18 years.
- participants with CAP<250 kpa<8kP by a FibroScan™ within the past 6 months.

### Exclusion criteria for the main study

- Have allergy toward soya, milk or chocolate.
- Have allergy toward pectin.
- Participants on a vegan diet.
- Have eating disorders or difficulties or gastrointestinal conditions e.g. malabsorptive conditions such as coeliac, irritable bowel syndrome (IBS) or Inflammatory Bowel Disease (IBD) or gastroparesis.
- Have chronic malnutrition condition.
- History of major surgery which potentially limits participation or completion of the study.
- History of previous intestinal surgery known to affect food intake
- or digestive function, including bariatric surgery.
- Use of antibiotics, antifungal medications, probiotics or prebiotics 90 days before the start of the study.
- Are taking the following medications: immunosuppressants, amiodarone and/or perhexiline.
- Are currently following or anticipated to commence a specialised commercially available weight loss diet and/or program or concomitant use of any weight loss medication or herbal weight loss products.
- History of side effects towards probiotics or prebiotics.
- History or current psychiatric illness.
- History or current neurological condition (e.g. epilepsy).
- Participants with other liver abnormalities.
- Evidence of monogenic metabolism diseases such as Lysosomal acid lipase deficiency (LALD), Wilson disease, Hypobetalipoproteinemia, or inborn errors of metabolism.
- Have had a weight change exceeding 3 kg within 3 months.
- Uncontrolled diabetes, active malignancy, or chronic infections.
- Having symptoms of active infection.
- Excessive alcohol intake defined as self-reported intakes greater than 21 units per week in men, and 14 units per week in women.
- Participants who are pregnant, breast feeding or actively planning pregnancy will be excluded from the study.
- Participation in any other trial in the last 3 months.

**Exclusion criteria for healthy volunteers MRI scans and patients optional MRI scans:**

- Contraindications for MRI scanning: having pacemakers, defibrillators, neurostimulators, prohibited medical implants, and foreign bodies (e.g. bullets, shrapnel, metal slivers), history of metallic foreign body in eye(s) and penetrating eye injury that could present a risk during an MRI scan.
- Difficulty breathing or inability to lie flat, as well as conditions that could worsen under stress (such as anxiety or panic disorders, claustrophobia, uncontrolled hypertension, or seizure disorders) severe enough to prevent undergoing an MRI.
- Contraindications to Hyoscine butylbromide (Buscopan®): Previous adverse drug reaction (allergic, hypersensitivity or other), Angle-closure glaucoma, tachycardia, ischaemic heart disease, myasthenia gravis, prostatic enlargement with urinary retention requiring catheterisation, mechanical stenosis in the gastrointestinal tract, paralytic or obstructive ileus, hypotension, cardiac disease, recent heart attack or any arrhythmias, thyrotoxicosis, gastro-oesophageal reflux disease, hiatus hernia, ulcerative colitis.

### Expected duration of participant participation

### Study participants will be participating in the study for 6 weeks, attending two 2 hours study visits. Participants who will agree to have MRI scans, including healthy volunteers, will need to attend two 4 hours MRI visits.

### Removal of participants from therapy or assessments/Participant Withdrawal

Participants may be withdrawn from the trial either at their own request or at the discretion of the Investigator due to safety reasons (including pregnancy) or development of severe adverse events, or due to failure to adhere to protocol requirements. The participants will be made aware that this will not affect their future care. Participants will be made aware (via the information sheet and consent form) that should they withdraw the data collected to date cannot be erased and may still be used in the final analysis. The research team will advise pregnant participants to stop the intervention and contact their GP if they have questions.

Study team will try their best to replace withdrawn participants.

### Informed consent

All study participants will provide written informed consent to a consent-trained researcher that will be taken in person at Nottingham University Hospitals NHS Trust during the screening visit.

Similarly, all healthy volunteers will provide written informed consent to a consent-trained researcher that will be taken in person at Sir Peter Mansfield Imaging Centre (SPMIC).

The Informed Consent Form will be signed and dated by the participant before they enter the trial. The researcher will explain the details of the trial and provide a Participant Information Sheet, ensuring that the participant has sufficient time to consider participating or not. The researcher will answer any questions that the participant has concerning study participation.

Informed consent will be collected from each participant before they undergo any interventions (including physical examination and history taking) related to the study. One copy of this will be kept by the participant, one will be kept by the researcher, and a third will be retained in the patient’s hospital records.

Should there be any subsequent amendment to the final protocol, which might affect a participant’s participation in the trial, continuing consent will be obtained using an amended Consent form which will be signed by the participant.

# TRIAL / STUDY INTERVENTION AND REGIMEN

Interested participants will be asked to contact the study team using the provided contact details on the advertisement poster or the study team will directly contact the potential participants if they are referred for recruitment by patient’s usual care team (which may include the investigator)at hepatology clinic via using encrypted emails (consent to share contact details will be documented in the patients notes) or those who have consented to contact for future research. The Participant Information Sheet (PIS) will be given to those expressing interest, either in person at NIHR Nottingham Clinical Research Facility at Nottingham University Hospitals NHS Trust, Queens Medical Centre or via royal mail or email. Study team contact information will be included in the PIS. Participants will have up to one week to decide whether they wish to take part or not. If a participant expresses interest after reading the PIS, they will be invited for a screening visit, which will be conducted in the NIHR Nottingham Clinical Research Facility at Nottingham University Hospitals NHS Trust, Queens Medical Centre by a trained member of the study team. During screening visit, the research team member will go through study details, allow the participant to ask any questions they have, and consider their decision. If they are willing to participate, they will be asked to provide consent and will be formally enrolled in the study. The research team member or member of the usual care team will then go through eligibility checklist with the participant to confirm their eligibility to participate in the study. If the medical notes are not available for participants to check their eligibility, then the chief investigator will rely on participants answers regarding their medications and medical conditions to confirm eligibility. A trained research Practitioner or nurse, who will be a member of research team at Nottingham University Hospital NHS Trust, will also perform CAP- FibroScan™ to measure liver fat and stiffness (if not completed in past month or not available in the medical note) and the result will be provided to the participant at the visit and will be documented in the medical notes. FibroScan™ results will be sent to the chief investigator to confirm eligibility.

FibroScan ™ is owned and maintained by Nottingham University Hospitals NHS Trust.

If chief investigator finds anything abnormal on FibroScan™ results, the investigator will arrange for an appropriately qualified doctor, from a healthcare provider e.g. a NHS Trust or a private doctor, to look at them. That specialist doctor would contact participants GP to explain the situation, so that GP could then advise the participant.

Due to FibroScan™ requirements, participants will be asked to stop eating 4 hours before the scan visit, stop drinking alcohol 24 hours before the scan visit and to stop drinking water 2 hours prior to the scan visit.

Research team will schedule an onsite baseline visit with eligible participant. Participants will be given a stool collection kit before scheduling their baseline visit. Stool kit will be given either in person or mailed to the participant.

If participant gave consent and were found not to be eligible, including FibroScan™ results, then the study team will not enrol them within the study.

All in person visits will be conducted at adult NIHR Nottingham Clinical Research Facility at Nottingham University Hospitals NHS Trust, Queens Medical Centre or at Sir Peter Mansfield Imaging Centre (SPMIC).

**Screening and recruitment flow diagram:**

**Hepatology Clinics/ Posters/email/**

**social media/ phone calls**

**Provide PIS**

**(visit/ email)/ provide invitation letter with PIS to referred patient (visit/ email)**

**Participant Consented and screened**

**Recruited into study**

*Invitation*

*Screening*

*Enrolment*

Contact study team/ referred to study team

Eligible participants

**Baseline measures (visit 1):**

Participants will attend the visit fasted for at least 6 hours and provide the stool samples. Anthropometric measurements, including weight, height, waist/hip ratio and blood pressure, will be taken in duplicate by a trained researcher using standard procedures. A trained researcher will collect a blood sample (not more than 40ml) for analysis. Moreover, if participants consent to genetic testing, then blood sample for genetic analysis will be taken too. After randomisation, participants will receive their assigned pre-weighed supplement in food-grade pouches.

All the research activities at baseline visit will be done by Noor Al-Tameemi except blood and stool samples collection that will be done by a trained research practitioner or nurse, who will be a member of research team at Nottingham University Hospital NHS Trust.

**MRI visit 1 and 2:**

If the participant consents to have MRI scans, then the study team will schedule a scan for them within the same day of baseline visit. Both MRI scans visits will be arranged at the Sir Peter Mansfield Imaging Centre (SPMIC). The participant will attend the visit fasted and provide the stool sample. The MRI research team will use Philips 3T MRI scanner. The team will firstly scan the hepatic fat and other surrounding GI organs fat using Dixon MRI sequences. Then, a medical doctor will cannulate the participant, take a blood sample from the participant and will ask them to drink the oral contrast which consists of 2.5% Mannitol and 0.2% Locust Bean Gum and will be prepared at the SPMIC on the morning of the scan. The participant will be asked to drink the oral contrast gradually over 40 minutes. This contrast required to provide contrast between the lining of the gut and its contents. After that, the medical doctor will prescribe and administer hyoscine butylbromide (Buscopan®) through the cannula while MRI research team taking T2* MRI measures of gut wall to measure gut permeability. Buscopan® is used to stop the bowel moving to allow the team to generate clear pictures of the bowel wall.

The medical doctor will confirm eligibility and document this, prior to performing any research activity related to the cannulation/administration of hyoscine butylbromide (Buscopan®).

Both MRI and baseline visit will take around 4 hours to complete all the research activities.

It is extremely unlikely that MRI scan will show any abnormality. Even if there were an abnormality, it is unlikely that research team would notice it since we are taking these MRI scans for scientific research, so they are not the same as scans collected by doctors for medical purposes. Furthermore, the pictures will not be looked at by a radiologist.

If the research team did find anything abnormal on the MRI scan, the investigator would arrange for an appropriately qualified doctor, from a healthcare provider e.g. a NHS Trust or a private doctor, to look at them. That specialist doctor would contact participant GP to explain the situation, so that participant GP could then advise them.

**Dietary intervention (6 weeks):**

Control and intervention arms: Each participant will be randomly assigned to either pectin with whey protein and cocoa powder or control whey protein with cocoa powder using online software (sealedenvelope.com) to determine intervention A or B at enrolment. Randomisation will ensure equal distribution of key characteristics that might confound between-group comparisons and will be evaluated using baseline data analysis. Participants in the intervention arm (N=15) will be required to take 15g of pectin combined with 15g of unflavoured whey protein powder and 5g of cocoa powder as a flavour per day for a period of 6 weeks. The participants in the placebo/control arm (N=15) will be required to take 30g of unflavoured whey protein powder combined with 5g of cocoa powder as a flavour per day for the same period of 6 weeks. All supplements will be provided, pre-weighed, in food grade pouches and will be given sufficient cover the total intake for the 6-weeks period. Participants will be asked to incorporate the supplement provided in 250-300ml of water taken once at morning or evening. Regular weekly contact will be made via phone calls by Noor Al-Tameemi to record patients’ feedback or any adverse event that might occur due to ingestion of supplements and to ensure participants receive any information that becomes available during the course of the research that may be relevant to their continued participation .

**Follow-up/end of study measures (visit 2)**

At the end of 6 weeks, as for visit 1, stool samples and fasting blood samples (not more than 40ml), including blood sample for genetic analysis if the participants consented to that, will be collected, weight, height, waist/hip ratio and blood pressure measured, and FibroScan™ performed. The last visit will last no longer than 2 hours.

All the research activities at follow-up visit will be done by Noor Al-Tameemi except blood and stool samples collection and FibroScan™ that will be done by a trained research practitioner or nurse, who will be a member of research team at Nottingham University Hospital NHS Trust.

If the participant agrees to have MRI scans, then the study team will schedule a follow-up MRI scan as for MRI visit 1, taking around 4 hours.

**Schedule of events**

|  | Screening | Visit 1 Baseline visit  (Day 1) | Visit 2 (6 weeks after visit 1) |
| --- | --- | --- | --- |
| Assessing participants eligibility and informed consent | X |  |  |
| Fasting blood sample collection |  | X | x |
| Faecal sample (collection by participant at home & brought to visit) |  | X | x |
| Anthropometric measurements (weight, height, waist/hip ratio and blood pressure) |  | X | x |
| CRF completion (demographics, health/med history) |  | x | x |
| Randomisation |  | X |  |
| Dietary supplementation Intervention start (dispense supplements and instruct) |  | x |  |
| FibroScan™ | x |  | x |
| MRI screening questionnaire (optional) |  | x | x |
| MRI (optional) |  | x | x |

**Healthy Volunteers MRI Scans**

Both healthy volunteers MRI scans visits will be arranged at the Sir Peter Mansfield Imaging Centre (SPMIC). The participant will attend the visit fasted. The MRI research team will use Philips 3T MRI scanner. The team will firstly scan the hepatic fat and other surrounding GI organs fat using Dixon MRI sequences. Then, a medical doctor, who is a member of the University of Nottingham research ream, will cannulate the participant, take a blood sample from the participant and will ask them to drink the oral contrast which consists of 2.5% Mannitol and 0.2% Locust Bean Gum and will be prepared by MRI research team at the SPMIC on the morning of the scan. The participant will be asked to drink the oral contrast gradually over 40 minutes. This contrast required to provide contrast between the lining of the gut and its contents. After that, the medical doctor will prescribe and administer hyoscine butylbromide (Buscopan®) through the cannula while MRI research team taking T2* MRI measures of gut wall to measure gut permeability. Buscopan® is used to stop the bowel moving to allow the team to generate clear pictures of the bowel wall.

The medical doctor will confirm eligibility and document this, prior to performing any research activity related to the cannulation/administration of hyoscine butylbromide (Buscopan®).

Each MRI scans visit will take around 4 hours.

It is extremely unlikely that MRI scan will show any abnormality. Even if there were an abnormality, it is unlikely that research team would notice it since we are taking these MRI scans for scientific research, so they are not the same as scans collected by doctors for medical purposes. Furthermore, the pictures will not be looked at by a radiologist.

If the research team did find anything abnormal on the MRI scan, the investigator would arrange for an appropriately qualified doctor, from a healthcare provider e.g. a NHS Trust or a private doctor, to look at them. That specialist doctor would contact participant GP to explain the situation, so that participant GP could then advise them.

### Compliance

No interim data analyses are planned and given the simplicity of the study we will not need a data monitoring committee nor the overview of a formal study steering committee. The participant’s involvement in the study will only be terminated if the participant wishes to do so or if participants or caring clinician feel that the participants are too unwell to carry on with the study.

The study will be terminated if the participant safety is deemed to be at risk throughout the study.

### Criteria for terminating trial

Stopping the trial is unexpected however might be considered if a major safety concern will be reported or due to poor recruitment or loss of resources. In such case, unused supplements stock will be disposed as food waste into landfill waste.

Research data will be archived based on the archiving plan.

## TRANSPORT AND STORAGE OF THE TISSUES

Samples will be stored in a linked anonymised format and labelled using a combination of study reference, unique study identifier and cross referenced with location code numbers to permit accurate linkage to study data and the consent form. Where participant’s consent, samples will be collected for analysis of common genetic variants associated with development of gut permeability [15].

Stool samples will be collected and stored in aliquots at -80°C.

Blood samples will be collected and either sent for clinical analyses or stored or processed to obtain plasma and serum aliquots which will be stored at -80°C. They will be transported within the building from the patient to the lab in double containment with absorbent material and following Biosafety requirements.

The master sample database will be held by the research team in a password encrypted file.

The analysis of samples will take place at the University of Nottingham within the School of Medicine located in the Queens Medical Centre, or at Nottingham University Hospitals NHS Trust, Queens Medical Centre clinical laboratories or at contracted commercial service providers.

Samples will be transferred by an approved courier for analysis by external agents at the end of the study. All shipments will contain a complete inventory of all samples, along with the name of the person responsible for sending the samples. Any remaining samples at the University of Nottingham will be stored either under the University HTA Licence (DI James Dixon- Licence Number 12265) or within a Research Tissue Bank for future research if participants are agreeable and sign the optional clause on the consent form.

Where participants do not agree to the future use of the samples they will be destroyed after the study ends in accordance with the Human Tissue Act, 2004.

## LABORATORY ANALYSES

Microbial DNA will be extracted from stool samples by the research laboratory team and sent for specialist sequencing analysis to obtain the corresponding DNA sequences. From this we can determine the bacterial species present based on their characteristic DNA signatures.

Blood biomarkers and metabolites will be analysed by the research laboratory team using commercial assay kits or sent for specialist analytics by contracted commercial laboratories. Genetic variants such as MUC2 will be determined where consent for genetic analysis is given.

The laboratory work by the research team will take place in the School of Medicine Labs in the Queens Medical Centre which are centrally managed. All equipment is appropriately calibrated, maintained and serviced.

# STATISTICS

### Methods

The data will be analysed by Noor Al-Tameemi on-site at the University of Nottingham under supervision of the CI, co-investigators and NIHR Nottingham Biomedical Research Centre liver/GI statistical team.

All analyses will be conducted on password-protected University of Nottingham computers or University of Nottingham laptops, that are backed up to cloud servers.

Data input, cleaning and analysis will be conducted using a statistical software such as PRISM or SPSS (IBM Corp, Armonk, New York) or R software (R Foundation for Statistical Computing, Vienna, Austria). Descriptive statistics will be reported as means ± standard deviations (SD) or medians with interquartile ranges (IQR) for continuous variables, and as frequencies and percentages for categorical variables. Prior to analysis, data will be checked for normality using the Shapiro-Wilk test. Standard parametric and non-parametric tests (where assumptions of normality are violated) will be used to assess significance of changes compared to baseline Characteristics of participants, outcome measures at baseline (symptoms, clinical parameters) and post-intervention will be summarized by intervention group using descriptive statistics. The differences between the two groups will be tested by matched-pair Student’s t-test. Data will be presented as mean± standard deviation and the significance of difference will be set at p <0.05.

Mixed-effects linear regression models will be used to assess the intervention effect of pectin compared to placebo, adjusting for baseline values and relevant covariates such as age, sex, ethnicity and BMI. The interaction between time (baseline and follow-up) and group (pectin vs placebo) will be examined to determine if the changes over time differ between the two groups.

Any changes in the planned statistical methods will be documented in the study report.

### Sample size and justification

The sample size was calculated using Raosoft® calculator [35] with Confidence level of 95% ± 5% and response distribution of (50%) to know the minimum sample size. The total sample size came out to be 14 per arm. The sample size of this study is equal to 15 per arm to obtain the statistical power for the study.

This calculation was based on a previous pectin pilot study which found significant differences in 4 inflammatory markers with n=14 healthy adult volunteers [31].

### Assessment of efficacy

### Primary endpoint

Levels of serum inflammatory biomarkers such as; IL-6, IL-10, C-RP, Zonulin (Haptoglobulin), IL-1β, TNFα, IFNᵧ.

### Secondary endpoint

- Change in BMI
- Change in general metabolic indicators such as fasting blood glucose
- Changes in blood-based markers relevant to MASLD (e.g., CK18-M30, CK18-M65, PROC3, Enhanced Liver Fibrosis (ELF), NIS2+™, YKL-40, microRNA miR-34a-5p, liver-associated enzymes such as Alanine Aminotransferase (ALT), Aspartate Aminotransferase (AST), gamma-glutamyl transferase (GGT), Alkaline Phosphatase (ALP)), bilirubin levels, lipid profiles, and platelet counts.
- Gut microbiome: changes in composition
- Change in non-invasive physiological assessments linked to liver characteristics: FibroScan™ CAP value and Liver stiffness based on vibration-controlled transient elastography (VCTE)
- Change in liver fat and other surrounding abdominal organs fat through alterations in Dixon MRI sequence
- Alterations in T2* MRI measures of intestinal permeability

The efficacy parameters for the primary and secondary endpoints will involve calculating the difference between baseline visit and follow-up visit in both study arms and compare between two arms. Moreover, they will be used in calculating correlation coefficients (Pearson's or other appropriate methods) between the effect of pectin compared to placebo, adjusting for baseline values and relevant covariates such as age, sex, ethnicity and BMI. These correlation coefficients will indicate the strength and direction of the relationships between the variables.

Primary endpoint has been identified based on the results on a recent study that was conducted at University of Nottingham, showed that 4 weeks ingestion of 15-20g/day of LM pectin in healthy volunteers significantly reduced pro-inflammatory blood markers [31].

### Assessment of safety

This study is not aimed at primarily assessing safety as the study is a dietary intervention using components sourced from common foods, research team foresee no adverse reaction or side effects. Increasing fibre intake may result in bloating amongst those who have a low habitual intake of dietary fibre. However, such effects are mild and not considered as a safety concern. Those reporting having inflammatory bowel disease, those who experience irritable bowel syndrome, and those having had gastrointestinal resection surgery will not be recruited. Moreover, those with food allergies or sensitivities to components of the investigational products will not be recruited.

Pregnant and breastfeeding women will be excluded from the study because there is limited data regarding the safety of pectin and whey protein consumption during on pregnancy and breastfeeding.

The common side effect from administration of the Buscopan® (that will be used during MRI scans as antispasmodics) are very short lived (blurred vision and raised heart rate) and participants will be advised to stay at the test centre until their vision returns to normal (this normally occurs within 30 minutes of the injection). Rare side effects from administration of the Buscopan® injection include skin reactions, tachycardia, dry mouth, dyshidrosis, and in extremely rare cases angle-closure glaucoma. This will be monitored throughout the study visit at Sir Peter Mansfield Imaging Centre (SPMIC) by a registered medical doctor who will be a member of the research team and who will administer Buscopan® and monitor participants.

Mannitol 2.5% / Locust bean gum 0.2% will be used during MRI scans as oral contrast. Mannitol increases MRI contrast between the lumen and the bowel wall, and the addition of locust bean gum serves to counteract some of the recognised side effects associated with Mannitol ingestion, which can include flatulence and alteration of gastrointestinal microbiota leading to bowel spasms and diarrhoea.

### Procedures for missing, unused and spurious data

Every effort will be made within the study to minimise the occurrence of missing data. Where possible, all data available will be used.

### Definition of populations analysed

Safety set: All randomised participants who receive at least one intervention.

Full Analysis set: All randomised participants, who participated in at least one intervention and for whom at least one post-baseline assessment of the primary endpoint is available.

Per protocol set: All participants in the Full Analysis set who are deemed to have no major protocol violations that could interfere with the objectives of the study.

# ADVERSE EVENTS

### Definitions

**An adverse event is any unfavourable and unintended sign, symptom, syndrome or illness that develops or worsens during the period of observation in the study.**

An AE does include a / an:

1. exacerbation of a pre-existing illness.

2. increase in frequency or intensity of a pre-existing episodic event or condition.

3. condition detected or diagnosed after medicinal product administration even though it may have been present prior to the start of the study.

4. continuous persistent disease or symptoms present at baseline that worsen following the start of the study.

An AE does not include a / an:

1. medical or surgical procedure (e.g., surgery, endoscopy, tooth extraction, transfusion); but the condition that lead to the procedure is an AE.

2. pre-existing disease or conditions present or detected at the start of the study that did not worsen.

3. situations where an untoward medical occurrence has not occurred (e.g., hospitalisations for cosmetic elective surgery, social and / or convenience admissions).

4. disease or disorder being studied or sign or symptom associated with the disease or disorder unless more severe than expected for the participant’s condition.

5. overdose of concurrent medication without any signs or symptoms.

A Serious Adverse Event (SAE) is any adverse event occurring following study mandated procedures, having received the intervention that results in any of the following outcomes:

1. Death

2. A life-threatening adverse event

3. Inpatient hospitalisation or prolongation of existing hospitalisation

4. A disability / incapacity

5. A congenital anomaly in the offspring of a participant

Important medical events that may not result in death, be life-threatening, or require hospitalisation may be considered a serious adverse event when, based upon appropriate medical judgment, they may jeopardize the patient or participant and may require medical or surgical intervention to prevent one of the outcomes listed in this definition

Since the current study is a dietary intervention, research team do not foresee any adverse events. However, if the participant developed a severe adverse reaction to taking the allocated fibre supplement (i.e. severe bloating amongst those who have a low habitual intake of dietary fibre), they will be asked to contact the study team immediately. Based on the severity of the event and at the discretion of the CI, the study team may advise the participant to continue or withdraw from the study.

All adverse events will be assessed for seriousness, expectedness and causality:

A distinction is drawn between serious and severe AEs. Severity is a measure of intensity whereas seriousness is defined using the criteria above. Hence, a severe AE need not necessarily be serious.

### Causality

**Not related or improbable**: a clinical event including laboratory test abnormality with temporal relationship to trial intervention administration which makes a causal relationship incompatible or for which other treatments, chemicals or disease provide a plausible explanation**.** This will be counted as “unrelated” for notification purposes.

**Possible**: a clinical event, including laboratory test abnormality, with temporal relationship to trial intervention administration which makes a causal relationship a reasonable possibility, but which could also be explained by other interventions, chemicals or concurrent disease. This will be counted as “related” for notification purposes.

**Probable**: a clinical event, including laboratory test abnormality, with temporal relationship to trial intervention administration which makes a causal relationship a reasonable possibility, and is unlikely to be due to other interventions, chemicals or concurrent disease. This will be counted as “related” for notification purposes.

**Definite**: a clinical event, including laboratory test abnormality, with temporal relationship to trial intervention administration which makes a causal relationship a reasonable possibility, and which can definitely not be attributed to other causes. This will be counted as “related” for notification purposes.

With regard to the criteria above, medical and scientific judgment shall be used in deciding whether prompt reporting is appropriate in that situation.

### Reporting of adverse events

Participants will be asked to contact the study site immediately in the event of any serious adverse event. All adverse events will be recorded and closely monitored until resolution, stabilisation, or until it has been shown that the study intervention is not the cause. The Chief Investigator shall be informed immediately of any serious adverse events and shall determine seriousness and causality in conjunction with any treating medical practitioners.

##

## Trial Intervention Related SAEs

**A serious adverse event that is unexpected in its severity and seriousness *and* deemed directly related to or suspected to be related to the trial intervention shall be reported to the ethics committee that gave a favourable opinion as stated below.**

**The event shall be reported immediately of knowledge of its occurrence to the Chief Investigator.**

**The Chief Investigator will:**

- Assess the event for seriousness, expectedness and relatedness to the trial treatment or intervention.
- Take appropriate medical action, which may include halting the trial and inform the Sponsor of such action.
- If the event is deemed related to the trial intervention shall inform the REC using the reporting form found on the NRES web page within 7 days of knowledge of the event.
- Shall, within a further eight days send any follow-up information and reports to the REC.
- Make any amendments as required to the study protocol and inform the REC as required

### Participant removal from the study due to adverse events

Any participant who experiences an adverse event may be withdrawn from the study at the discretion of the Investigator.

# ETHICAL AND REGULATORY ASPECTS

## ETHICS COMMITTEE AND REGULATORY APPROVALS

The trial will not be initiated before the protocol, informed consent forms and participant and GP information sheets have received approval / favourable opinion from the Research Ethics Committee (REC), the respective National Health Service (NHS) or other healthcare provider’s Research & Development (R&D) department, and the Health Research Authority (HRA) if required. Should a protocol amendment be made that requires REC approval, the changes in the protocol will not be instituted until the amendment and revised informed consent forms and participant information sheets have been reviewed and received approval / favourable opinion from the REC and R&D departments. A protocol amendment intended to eliminate an apparent immediate hazard to participants may be implemented immediately providing that the REC are notified as soon as possible, and an approval is requested. Minor protocol amendments only for logistical or administrative changes may be implemented immediately; and the REC will be informed.

The trial will be conducted in accordance with the ethical principles that have their origin in the Declaration of Helsinki, 1996; the principles of Good Clinical Practice, and the UK Department of Health Policy Framework for Health and Social Care, 2017.

## INFORMED CONSENT AND PARTICIPANT INFORMATION

The process for obtaining participant informed consent will be in accordance with the REC guidance, and Good Clinical Practice (GCP) and any other regulatory requirements that might be introduced. The investigator or their nominee and the participant shall both sign and date the Informed Consent Form before the person can participate in the study.

The participant will receive a copy of the signed and dated forms and the original will be retained in the Trial Master File. A second copy will be filed in the participant’s medical notes and a signed and dated note made in the notes that informed consent was obtained for the trial.

The decision regarding participation in the study is entirely voluntary. The investigator or their nominee shall emphasize to them that consent regarding study participation may be withdrawn at any time without penalty or affecting the quality or quantity of their future medical care, or loss of benefits to which the participant is otherwise entitled. No trial-specific interventions will be done before informed consent has been obtained.

The investigator will inform the participant of any relevant information that becomes available during the course of the study, and will discuss with them, whether they wish to continue with the study. If applicable they will be asked to sign revised consent forms.

If the Informed Consent Form is amended during the study, the investigator shall follow all applicable regulatory requirements pertaining to approval of the amended Informed Consent Form by the REC and use of the amended form (including for ongoing participants).

## RECORDS

### Case Report Forms

Each participant will be assigned a trial identity code number, allocated at randomisation if appropriate, for use on CRFs other trial documents and the electronic database. The documents and database will also use their initials (of first and last names separated by a hyphen or a middle name initial when available) and date of birth (dd/mm/yy).

CRFs will be electronic using REDCap secure system and will include participants anthropometric measurements, participants demographics and health/medication history, and FibroScan™ and MRI (if applicable) results.

CRFs will be treated as confidential documents and held securely in accordance with regulations. The investigator will make a separate confidential record of the participant’s name, date of birth, local hospital number or NHS number, and Participant Trial Number (the Trial Recruitment Log), to permit identification of all participants enrolled in the trial, in accordance with regulatory requirements and for follow-up as required.

CRFs shall be restricted to those personnel approved by the Chief or local Principal Investigator and recorded on the ‘Trial Delegation Log.’

All paper forms shall be filled in using black ballpoint pen. Errors shall be lined out but not obliterated by using correction fluid and the correction inserted, initialled and dated.

The Chief or local Principal Investigator shall sign a declaration ensuring accuracy of data recorded in the CRF.

With personnel who are qualified by education, training, and experience to perform their respective tasks and that the study will not use the services of study personnel for whom sanctions have been invoked or where there has been scientific misconduct or fraud.

Trial related information such as case report forms and other biochemical data will be stored in study files and updated to an electronic data base (Microsoft excel) to facilitate analyses. Electronic forms will be stored on UoN shared drives.

MRI data will be downloaded from the 3T UoN MRI machines and stored on UoN shared drives.

### Sample Labelling

Each participant will be assigned a trial identity code number for use on the samples, consent forms and other study documents and the electronic database. The documents and database will also use their initials (of first and last names separated by a hyphen or a middle name initial when available) and date of birth (dd/mm/yy).

Samples for NHS pathology analysis will be labelled in accordance with local NHS procedures.

### Source documents

Source documents shall be filed at the investigator’s site and may include but are not limited to, consent forms, current medical records, laboratory results and records. A CRF may also completely serve as its own source data. Only trial staff as listed on the Delegation Log shall have access to trial documentation other than the regulatory requirements listed below.

### Direct access to source data / documents

The CRF and all source documents, including progress notes and copies of laboratory and medical test results shall made be available at all times for review by the Chief Investigator, Sponsor’s designee and inspection by relevant regulatory authorities (e.g. DH, Human Tissue Authority).

## DATA PROTECTION

All trial staff and investigators will endeavour to protect the rights of the trial’s participants to privacy and informed consent, and will adhere to the Data Protection Act, 2018. The CRF will only collect the minimum required information for the purposes of the trial. Access to the information will be limited to the trial staff and investigators and relevant regulatory authorities (see above). Computer held data including the trial database will be held securely and password protected. All data will be stored on a secure dedicated web server. Access will be restricted by user identifiers and passwords (encrypted using a one-way encryption method).

Information about the trial in the participant’s medical records / hospital notes will be treated confidentially in the same way as all other confidential medical information.

Electronic data will be backed up every 24 hours to both local and remote media in encrypted format.

# QUALITY ASSURANCE & AUDIT

## INSURANCE AND INDEMNITY

Insurance and indemnity for trial participants and trial staff is covered within the NHS Indemnity Arrangements for clinical negligence claims in the NHS, issued under cover of HSG (96)48. There are no special compensation arrangements, but trial participants may have recourse through the NHS complaints procedures.

The University of Nottingham as research Sponsor indemnifies its staff with both public liability insurance and clinical trials insurance in of claims made by research subjects.

## TRIAL CONDUCT

Trial conduct may be subject to systems audit of the Trial Master File for inclusion of essential documents; permissions to conduct the trial; Trial Delegation Log; CVs of trial staff and training received; local document control procedures; consent procedures and recruitment logs; adherence to procedures defined in the protocol (e.g. inclusion / exclusion criteria, correct randomisation, timeliness of visits); adverse event recording and reporting; accountability of trial materials and equipment calibration logs.

## TRIAL DATA

Monitoring of trial data shall include confirmation of informed consent; source data verification; data storage and data transfer procedures; local quality control checks and procedures, back-up and disaster recovery of any local databases and validation of data manipulation. The Trial Coordinator/Academic Supervisor, or where required, a nominated designee of the Sponsor, shall carry out monitoring of trial data as an ongoing activity.

Entries on CRFs will be verified by inspection against the source data. A sample of CRFs (10% or as per the study risk assessment) will be checked on a regular basis for verification of all entries made. In addition, the subsequent capture of the data on the trial database will be checked. Where corrections are required, these will carry a full audit trail and justification.

Trial data and evidence of monitoring and systems audits will be made available for inspection by REC as required.

## RECORD RETENTION AND ARCHIVING

In compliance with the ICH/GCP guidelines, regulations and in accordance with the University of Nottingham Research Code of Conduct and Research Ethics, the Chief or local Principal Investigator will maintain all records and documents regarding the conduct of the study. These will be retained for at least 7 years or for longer if required. If the responsible investigator is no longer able to maintain the study records, a second person will be nominated to take over this responsibility.

The Trial Master File and trial documents held by the Chief Investigator on behalf of the Sponsor shall be finally archived at secure archive facilities at the University of Nottingham. This archive shall include all trial databases and associated meta-data encryption codes.

## DISCONTINUATION OF THE TRIAL BY THE SPONSOR

The Sponsor reserves the right to discontinue this trial at any time for failure to meet expected enrolment goals, for safety or any other administrative reasons. The Sponsor shall take advice from the Trial Steering Committee and Data Monitoring Committee as appropriate in making this decision.

## STATEMENT OF CONFIDENTIALITY

Individual participant medical information obtained in this study are considered confidential and disclosure to third parties is prohibited with the exceptions noted above.

Participant confidentiality will be further ensured by utilising identification code numbers to correspond to intervention data in the computer files.

Such medical information may be given to the participant’s medical team and all appropriate medical personnel responsible for the participant’s welfare.

If information is disclosed during the study that could pose a risk of harm to the participant or others, the researcher will discuss this with the CI and where appropriate report accordingly.

Data generated in this trial will be available for inspection on request by the participating physicians, the University of Nottingham representatives, the REC, local R&D Departments and the regulatory authorities.

# PUBLICATION AND DISSEMINATION POLICY

The results of the study will be posted on the study report at ClinicalTrials.gov once the study team complete data analysis.

The clinical study report will be used for publication and presentation at scientific meetings and in peer-reviewed journals, in internal reports.

Results will be published in the Noor Al-Tameemi’s PhD thesis and in student presentations/dissertations. We will report the study findings to the funder as outlined in the funding agreement.

All data will be anonymised, no participants will be identified in any publications. Where appropriate for scrutiny of results in publications and data sharing, we will make fully anonymised datasets available to other researchers.

Summaries of results will also be made available to funders for dissemination. A lay summary will be available on the NIHR Nottingham BRC web site and associated Nottingham University Hospitals NHS Trust or University of Nottingham social media platforms and via direct contact with the CI. A summary will also be sent to participants who consented to future contact, as contact details were recorded in the research database.

# USER AND PUBLIC INVOLVEMENT

The NIHR Nottingham Biomedical Research Centre Digestive Diseases Patient Advisory group supported on-going studies investigating biomarkers of NAFLD where the need for practical interventions was highlighted. Members of the Patient Advisory group have provided guidance about the study proposal design and patient perspective considered in developing study procedures and participant documents through focus groups. Potential interventions were explored and discussed with a NAFLD patient group. Also, participants in an earlier study which used a similar study design but with an intervention of a low GI diet provided further insight into some challenges they face with maintaining substantial dietary changes and indicated that a simple supplement intervention would be preferred [36].

Our team also hosted an event to engage patients including workshops to determine potential unanswered research questions (2016) and provide feedback to the James Lind Alliance for priority setting. This research sets out to address some of these questions [37]. We held subsequent patient engagement events (May 2018) to seek input on refining the study plan and design and for increasing visibility and accessibility for patients to participate in research. We plan to consult the NIHR Nottingham BRC Patient Advisory Group in developing further materials for dissemination to patients and public. Patient advisors have also committed to support this study in the future.

Dissemination of the research findings to the patients and public will be through local in-person events such as open days and via live online public lectures and discussion forums (such as the Nottingham University Hospitals NHS TRUST ‘research lounge’ online August 2021 and December 2023). We will also provide progress updates directly to those who consent for future contact and to the Public on the NIHR Nottingham BRC web page and social media and local posters in UoN and Nottingham University Hospitals NHS Trust buildings.

# STUDY FINANCES

### Funding source

This study is funded by NIHR Nottingham BRC and via a PhD studentship to Noor Al-Tameemi.

###

### Participant stipends and payments

Participants will receive a £10 inconvenience allowance for their participation in the study at the end of the trial.

Participants who will agree to have 2 MRI visits will receive a £120 inconvenience allowance for their participation to be paid after completing the second MRI visit. Healthy volunteers who will agree to have 2 MRI visits will receive £120 inconvenience allowance for their participation to be paid after completing the second MRI visit.

If participants withdraw from the study for medical reasons not associated with the study, they will receive an inconvenience allowance proportional to the length of the period of participation, but if they withdraw for any other reason, the inconvenience allowance to be received, if any, shall be at the discretion of the investigator.

Travel and parking expenses (up to £10) will be offered for any visits incurred as a result of participation.

# REFERENCES

1. American Diabetes Association Professional Practice, C., *6. Glycemic Goals and Hypoglycemia: Standards of Care in Diabetes—2024.* Diabetes Care, 2023. **47**(Supplement_1): p. S111-S125.

2. Teng, M.L.P., et al., *Global incidence and prevalence of nonalcoholic fatty liver disease.* Clinical and molecular hepatology, 2023. **29**(1): p. 32-42.

3. Trust, B.L. *MASLD, NAFLD, and fatty liver disease,” British Liver Trust*. 2024 [cited 2024 11/10/2024]; Available from: <https://britishlivertrust.org.uk/information-and-support/liver-conditions/masld-nafld-and-fatty-liver-disease/>

4. Excellence, N.I.f.H.a.C. *Non-alcoholic fatty liver disease (NAFLD): Prevalence*. 2023 [cited 2024 11/10/2024]; Available from: <https://cks.nice.org.uk/topics/non-alcoholic-fatty-liver-disease-nafld/background-information/prevalence/>.

5. Chalasani, N., et al., *The diagnosis and management of non-alcoholic fatty liver disease: Practice Guideline by the American Association for the Study of Liver Diseases, American College of Gastroenterology, and the American Gastroenterological Association.* Hepatology (Baltimore, Md.), 2012. **55**(6): p. 2005-2023.

6. Caligiuri, A., A. Gentilini, and F. Marra, *Molecular pathogenesis of NASH.* International journal of molecular sciences, 2016. **17**(9): p. 1575-1575.

7. Jing, X., et al., *RG-I pectin-like polysaccharide from Rosa chinensis inhibits inflammation and fibrosis associated to HMGB1/TLR4/NF-κB signaling pathway to improve non-alcoholic steatohepatitis.* Carbohydrate polymers, 2024. **337**: p. 122139-122139.

8. Wang, S.-W., et al., *Liver and atherosclerotic risks of patients with cryptogenic steatotic liver disease.* Hepatology international, 2024. **18**(3): p. 943-951.

9. Rinella, M., et al., *A multi-society Delphi consensus statement on new fatty liver disease nomenclature.* Annals of hepatology, 2023.

10. Wei, S., et al., *NAFLD and NASH: etiology, targets and emerging therapies.* Drug discovery today, 2024. **29**(3): p. 103910-103910.

11. Jiang, S.Y., et al., *Discovery of an insulin‐induced gene binding compound that ameliorates nonalcoholic steatohepatitis by inhibiting sterol regulatory element‐binding protein–mediated lipogenesis.* Hepatology (Baltimore, Md.), 2022. **76**(5): p. 1466-1481.

12. Leung, C., et al., *The role of the gut microbiota in NAFLD.* Nature reviews. Gastroenterology & hepatology, 2016. **13**(7): p. 412-425.

13. Volynets, V., et al., *Nutrition, Intestinal Permeability, and Blood Ethanol Levels Are Altered in Patients with Nonalcoholic Fatty Liver Disease (NAFLD).* Digestive diseases and sciences, 2012. **57**(7): p. 1932-1941.

14. Vrieze, A., et al., *Transfer of Intestinal Microbiota From Lean Donors Increases Insulin Sensitivity in Individuals With Metabolic Syndrome.* Gastroenterology (New York, N.Y. 1943), 2012. **143**(4): p. 913-916.e7.

15. Liu, Y., et al., *The role of MUC2 mucin in intestinal homeostasis and the impact of dietary components on MUC2 expression.* International journal of biological macromolecules, 2020. **164**: p. 884-891.

16. Lu, P., et al., *Colonic gene expression patterns of mucin muc2 knockout mice reveal various phases in colitis development.* Inflammatory bowel diseases, 2011. **17**(10): p. 2047-2057.

17. Scott, R.A., et al., *MR Measures of Small Bowel Wall T2 Are Associated With Increased Permeability.* Journal of magnetic resonance imaging, 2021. **53**(5): p. 1422-1431.

18. (NICE), N.I.f.H.a.C.E. *Non-alcoholic fatty liver disease (NAFLD): assessment and management*. 2016 [cited 2024 12/11/2024].

19. Xu, X., J. Jin, and Y. Liu, *Performance of FibroScan in grading steatosis and fibrosis in patients with nonalcoholic fatty liver disease: A meta-analysis.* Arab journal of gastroenterology, 2023. **24**(4): p. 189-197.

20. Sundaram, T.S., et al., *Role of omega-3 polyunsaturated fatty acids, citrus pectin, and milk-derived exosomes on intestinal barrier integrity and immunity in animals.* Journal of animal science and biotechnology, 2022. **13**(1): p. 40-22.

21. Yang, X., Y. Yuan, and D. Xie, *Low Molecular Pectin Inhibited the Lipid Accumulation by Upregulation of METTL7B.* Applied biochemistry and biotechnology, 2021. **193**(5): p. 1469-1481.

22. Hu, W., A.M. Cassard, and D. Ciocan, *Pectin in Metabolic Liver Disease*. Nutrients, 2023. **15**(1): p. 157.

23. Li, W., K. Zhang, and H. Yang, *Pectin Alleviates High Fat (Lard) Diet-Induced Nonalcoholic Fatty Liver Disease in Mice: Possible Role of Short-Chain Fatty Acids and Gut Microbiota Regulated by Pectin.* Journal of agricultural and food chemistry, 2018. **66**(30): p. 8015-8025.

24. Houron, C., et al., *Gut microbiota reshaped by pectin treatment improves liver steatosis in obese mice.* Nutrients, 2021. **13**(11): p. 3725.

25. Delik, A., et al., *Metagenomic identification of gut microbiota distribution on the colonic mucosal biopsy samples in patients with non-alcoholic fatty liver disease.* Gene, 2022. **833**: p. 146587-146587.

26. Sun, Y., et al., *Low‐methoxyl lemon pectin attenuates inflammatory responses and improves intestinal barrier integrity in caerulein‐induced experimental acute pancreatitis.* Molecular nutrition & food research, 2017. **61**(4): p. np-n/a.

27. Wu, L., et al., *Anti-toll-like receptor 2 antibody ameliorates hepatic injury, inflammation, fibrosis and steatosis in obesity-related metabolic disorder rats via regulating MAPK and NF-κB pathways.* International immunopharmacology, 2020. **82**: p. 106368-106368.

28. Erridge, C., *Diet, commensals and the intestine as sources of pathogen-associated molecular patterns in atherosclerosis, type 2 diabetes and non-alcoholic fatty liver disease.* Atherosclerosis, 2011. **216**(1): p. 1-6.

29. Beukema, M., et al., *The impact of the level and distribution of methyl-esters of pectins on TLR2-1 dependent anti-inflammatory responses.* Carbohydrate polymers, 2021. **251**: p. 117093-117093.

30. Sahasrabudhe, N.M., et al., *Dietary fiber pectin directly blocks toll-like receptor 2-1 and prevents doxorubicin-induced ileitis.* Frontiers in immunology, 2018. **9**: p. 383.

31. Vijay, A., et al., *Supplementation with Citrus Low-Methoxy Pectin Reduces Levels of Inflammation and Anxiety in Healthy Volunteers: A Pilot Controlled Dietary Intervention Study.* Nutrients, 2024. **16**(19): p. 3326.

32. Xiong, R.-G., et al., *The Role of Gut Microbiota in Anxiety, Depression, and Other Mental Disorders as Well as the Protective Effects of Dietary Components.* Nutrients, 2023. **15**(14): p. 3258.

33. Ohlsson, L., et al., *Leaky gut biomarkers in depression and suicidal behavior.* Acta psychiatrica Scandinavica, 2019. **139**(2): p. 185-193.

34. tevens, B.R., et al., *Increased human intestinal barrier permeability plasma biomarkers zonulin and FABP2 correlated with plasma LPS and altered gut microbiome in anxiety or depression.* Gut, 2018. **67**(8): p. 1555-1557.

35. Raosoft. *Sample Size*. 2004 [cited 2024 12/11/2024].

36. Al-Awadi, A., *Assessment of dietary factors in patients with nonalcoholic fatty liver disease and an intervention study to investigate the effects of low glycaemic index diet*. 2023.

37. Gurusamy, K.S., et al., *Top research priorities in liver and gallbladder disorders in the UK.* BMJ open, 2019. **9**(3): p. e025045-e025045.
