# Supplementary material for: The impact of pectin supplementation on systemic inflammation pathways, gut microbiome, and metabolic health in patients with Metabolic Dysfunction-Associated Steatotic Liver Disease (MASLD): A study protocol for a randomised controlled trial
Source: PLoS One. 2026 Jul 13;21(7):e0352397. doi: 10.1371/journal.pone.0352397 (PMC13362096; doi:10.1371/journal.pone.0352397)
Supplement: Attachment — Submitted filename: Respond to editors and reviewers.docx [file pone.0352397.s005.docx]

**Response to Editor and Reviewers**

Manuscript ID: PONE-D-26-17120

Title: The impact of pectin supplementation on systemic inflammation pathways, gut microbiome, and metabolic health in patients with Metabolic Dysfunction-Associated Steatotic Liver Disease (MASLD): A study protocol for a randomised controlled trial

Dear Editor and Reviewers,

We sincerely thank the Academic Editor and Reviewers for their careful evaluation of our manuscript and for their constructive comments. We have revised the manuscript accordingly and believe the changes have substantially strengthened the protocol. All revisions have been incorporated into the revised manuscript with tracked changes.

Below, we provide a detailed point-by-point response to each comment.

**Responses to the Academic Editor**

**Comment 1**

**Please revise the term methoxyl instead of methoxy.**

Response:
We thank the Editor for this observation. The terminology has been revised throughout the manuscript from “methoxy” to “methoxyl” where appropriate to align with accepted pectin chemistry nomenclature.

*Changes were made throughout the manuscript.*

**Comment 2**

**Please revise the dose of pectin recommended by the manufacturer. Some recommend reconstituting 3.5–5 g/day by its consistency and osmotic load to treat constipation.**

Response:
We appreciate this important comment. The selected dose of 15 g/day was based on previously published human supplementation studies demonstrating acceptable tolerability and measurable biological effects at doses ranging from 15–20 g/day of low-methoxyl pectin. Furthermore, the intervention dose was developed in consultation with dietetic specialists during protocol development and was considered appropriate within the context of habitual fibre intakes that are typically below recommended levels in the general population. Current nutritional guidance recommends a daily dietary fibre intake of approximately 25–30 g/day for adults. In addition, the intervention is intended to investigate mechanistic metabolic and inflammatory effects rather than constipation treatment. Participants will be monitored throughout the study for gastrointestinal symptoms, and exclusion criteria were designed to minimise risks among individuals with pre-existing gastrointestinal disorders. Additional justification for dose selection and safety monitoring has now been added to the manuscript.

*Revisions added to the Introduction and Safety Considerations sections.*

**Comment 3**

**Please explain why in healthy adults, inflammatory cytokines were augmented and anti-inflammatory IL-10 diminished.**

Response:
We thank the Editor for highlighting the need for clarification. The cited pilot study reported reductions in pro-inflammatory cytokines together with an increase in the anti-inflammatory cytokine IL-10 following LM pectin supplementation. The manuscript has now been revised to improve clarity and to briefly describe the proposed mechanistic pathways underlying these immunomodulatory effects, including microbiota-mediated short-chain fatty acid production and improved gut barrier function.

*The relevant section in the Introduction has been revised for clarity and accuracy.*

**Comment 4**

**Please revise any literature about appropriate outcomes for clinical trials, establishing those relevant for patients and prioritized by core outcome set (COS). Consider the clinical over statistical relevance.**

Response:
We appreciate this valuable suggestion. Additional rationale has now been included to justify the selected outcomes based on mechanistic relevance to MASLD progression, inflammation, metabolic dysfunction, and patient-centred non-invasive disease assessment. We also clarified that the study is exploratory and hypothesis-generating, aiming to inform future larger clinical trials and core outcome development in MASLD nutritional interventions.

*Additional text has been incorporated into the Outcomes and Discussion sections.*

**Comment 5**

**Please explain and COS-based, why MRI is optional, not mandatory.**

Response:
We thank the Editor for this important point. MRI assessments were included as an optional sub-study due to logistical considerations, participant burden, scanner availability, and MRI contraindications that may limit participation. The primary and secondary outcomes of the trial rely on biochemical, anthropometric, FibroScan™, and clinical measures collected from all participants. MRI-derived measures are exploratory mechanistic outcomes intended to provide additional insight into hepatic fat distribution and intestinal permeability.

*Clarification has been added to the Methods section.*

**Comment 6**

**Please show the sample size calculation.**

Response:
We thank the Editor for this comment. The sample size calculation has now been expanded and clarified in the Statistical Analysis subsection, including assumptions regarding effect size, variability estimates, significance level, statistical power, and anticipated dropout rate.

*The Statistical Analysis section has been revised accordingly.*

**Comment 7**

**Complete in appropriate manner the tables (revise bullets, spaces, and other details).**

Response:
We appreciate this observation. All tables and formatting details have been carefully revised for consistency with PLOS ONE formatting requirements.

*Formatting corrections were made throughout the manuscript.*

**Comment 8**

**Since my point of view, I consider that you need a pilot study with dose acceptability by participants.**

Response:
We appreciate this suggestion. The intervention dose and study procedures were informed by previously published human studies evaluating LM pectin supplementation as well as prior dietary intervention studies conducted by the research team using similar methodologies. Based on these existing data and the low-risk nature of the intervention, an additional pilot acceptability study was not considered essential before conducting the present mechanistic trial.

**Additional Editorial Requirements**

**Clinical Trial Registration**

Response:
The Methods section has been updated to clarify that trial registration occurred after recruitment initiation due to administrative delays associated with completion of institutional and registry documentation processes. We confirm that the study protocol, outcomes, and analysis plan were predefined before participant enrolment.

The following statement has also been added:

“The authors confirm that all ongoing and related trials for this intervention are registered.”

**Funding Statement**

Response:
This study was funded by the National Institute for Health and Care Research (NIHR) Nottingham Biomedical Research Centre (award IS-BRC-1215-20003). The funders had no role in study design, data collection and analysis, decision to publish, or preparation of the manuscript.

No commercial entity had any role in the design, conduct, analysis, interpretation of data, or writing of this study.

**Competing Interests**

Response:
GPA has received consulting fees from Amryth, Agios, DNDi, PureTech LYT Inc, Pfizer Inc, GlaxoSmithKline, Clinipace, Merck Healthcare KGaA, JnJ, Suzhou MDCE Co Ltd, SynOx Therapies, Novartis Pharma, AstraZeneca and BenevolentAI Bio, paid to the University of Nottingham, and has received investigator-led research funding from Ipsen Ltd and Pfizer Inc. AMV is affiliated with commercial entities not related to the present study. Specifically, AMV is a member of the Scientific Advisory Board of Chuckling Goat and OLIPOP Inc. These roles are unrelated to the present study and had no influence on study design, data collection and analysis, decision to publish, or preparation of the manuscript. All other authors declare that the research was conducted in the absence of any commercial or financial relationships that could be construed as a potential conflict of interest. This does not alter our adherence to PLOS ONE policies on sharing data and materials.

**Ethics Statement**

Response:
The ethics statement has been retained only within the Methods section in accordance with journal requirements.

**Protocol File and Logos**

Response:
A clean version of the protocol with institutional logos and confidentiality notices removed has been uploaded in accordance with journal policy.

**Responses to Reviewer #1**

**Comment 1**

**The sample size calculation needs to be checked. It appears that the prior SD assumption seems to be the same for both groups.**

Response:
We thank the reviewer for this important observation. The sample size calculation has been revisited using unequal variance assumptions based on the referenced pilot data. The MRI sub-study sample size calculation was based on previously published quantitative MRI research evaluating changes in small bowel permeability following indomethacin provocation in healthy volunteers, which reported different standard deviations between groups (0.115 ± 0.063 s vs 0.070 ± 0.036 s). Therefore, unequal variance assumptions were used in the calculation. Recalculation did not materially alter the planned recruitment target. The sample size justification has now been clarified in the revised manuscript.

*Revisions added to the Statistical Analysis section.*

**Comment 2**

**The MAR assumption should have more justification. A retention strategy should also be included.**

Response:
We appreciate this helpful suggestion. Additional justification regarding the missing-at-random assumption has now been included. Furthermore, we added details regarding participant retention strategies, including regular participant contact, flexible scheduling, reminder communications, and reimbursement arrangements intended to minimise missing data and improve adherence.

*Additional text added to the Statistical Analysis and Study Procedures sections.*

**Comment 3**

**The use of a multivariate non-parametric alternative should be detailed in the case of non-normality.**

Response:
We thank the reviewer for this recommendation. The statistical analysis section has been revised to clarify that, where substantial deviation from normality persists despite transformation, appropriate non-parametric longitudinal or rank-based sensitivity analyses will be considered.

*Clarification added to the Statistical Analysis section.*

**Comment 4**

**Multiple comparison adjustment should be considered.**

Response:
We appreciate this important point. The manuscript has now been revised to clarify that secondary and exploratory analyses will be interpreted cautiously, and appropriate multiplicity adjustment approaches, including false discovery rate correction where appropriate, will be considered.

*Additional text added to the Statistical Analysis section.*

**Responses to Reviewer #2**

**General Comment**

**The rationale for dietary fibre intake levels and lack of dietary fibre intake data from daily meals are major concerns.**

Response:
We thank the reviewer for these important observations. We agree that habitual dietary fibre intake may influence study outcomes. At baseline, participants will complete study case report forms (CRFs) that include questions regarding current probiotic use and habitual dietary fibre intake. This information will be used to characterise participants’ baseline dietary patterns and identify major changes during the intervention period. In addition, population dietary intake data indicate that average fibre consumption in the general population is commonly below the recommended intake range of 25–30 g/day. The selected intervention dose was therefore designed to remain within a physiologically achievable dietary range while enabling assessment of mechanistic effects. The intervention dose was also developed in consultation with dietetic specialists during study design.

*Additional details have been added to the Outcome Assessments and Study Procedures sections.*

**Comment 1**

**A daily dose of 15 g of LM pectin may be excessive. Please provide the basis for determining this dosage. The issue of diarrhea must also be taken into account.**

Response:
We appreciate this important comment. The selected dose was informed by previous human supplementation studies demonstrating acceptable tolerability and measurable biological effects at doses ranging from 15–20 g/day of LM pectin. Participants with gastrointestinal disorders or sensitivities are excluded, and gastrointestinal symptoms will be monitored throughout the intervention. Safety considerations regarding bloating, gastrointestinal discomfort, and diarrhoea have now been further clarified.

*Additional rationale added to the Introduction and Safety Considerations sections.*

**Comment 2**

**The placebo formulation must also contain the same amount of dietary fibre.**

Response:
We thank the reviewer for this thoughtful suggestion. The placebo was intentionally designed without fermentable fibre to isolate the mechanistic effects of LM pectin supplementation on inflammatory and microbiome-related pathways. Inclusion of alternative fibre sources, including cellulose, may independently influence gastrointestinal physiology, microbial composition, and metabolic outcomes, thereby introducing additional confounding effects. The placebo was therefore selected to match flavour, appearance, and mode of administration while minimising biological activity.

*This rationale has now been clarified in the Intervention and Comparator section.*

**Comment 3**

**It is essential to examine dietary fibre intake from daily food records.**

Response:
We fully agree with the reviewer. At baseline, participants will complete study case report forms (CRFs) that include questions regarding current probiotic use and habitual dietary fibre intake. This information will be used to characterise participants’ baseline dietary patterns and identify major changes during the intervention period.

*Additional methodological details have been added to the manuscript.*

**Specific Comment**

**Please provide evidence that HM pectin has low fermentability.**

Response:
We thank the reviewer for this clarification. The manuscript wording has been revised to avoid overstatement. We now state that HM pectin may demonstrate lower fermentability relative to LM pectin depending on degree of esterification and microbial accessibility, supported by the relevant literature.

*The Introduction section has been revised accordingly.*

We sincerely thank the Editor and Reviewers again for their thoughtful and constructive comments, which have greatly improved the quality and clarity of our manuscript.

Sincerely,
Noor Kifah Al-Tameemi
On behalf of all authors
