# Supplementary material for: The impact of pectin supplementation on systemic inflammation pathways, gut microbiome, and metabolic health in patients with Metabolic Dysfunction-Associated Steatotic Liver Disease (MASLD): A study protocol for a randomised controlled trial
Source: PLoS One. 2026 Jul 13;21(7):e0352397. doi: 10.1371/journal.pone.0352397 (PMC13362096; doi:10.1371/journal.pone.0352397)
Supplement: Peer review history

# Peer review: Decision Letter 0

**DOI:** 10.1371/journal.pone.0352397.r001

**Authors:** Sophia Martínez-Vázquez

19 May 2026

PONE-D-26-17120

The impact of pectin supplementation on systemic inflammation pathways, gut microbiome, and metabolic health in patients with Metabolic Dysfunction-Associated Steatotic Liver Disease (MASLD): A study protocol for a randomised controlled trial

PLOS One

Dear Dr. Noor Kifah Al-Tameemi,

Thank you for submitting your manuscript to PLOS ONE. After careful consideration, we feel that it has merit but does not fully meet PLOS ONE’s publication criteria as it currently stands. Therefore, we invite you to submit a revised version of the manuscript that addresses the points raised during the review process.

==============================

Dear authors,

I revised carefully the protocol "The impact of pectin supplementation on systemic inflammation pathways, gut microbiome, and metabolic health in patients with Metabolic Dysfunction-Associated Steatotic Liver Disease (MASLD): A study protocol for a randomised controlled trial" and listed my comments centered mainly in methodological aspects:

1. Please revise the term methoxyl instead of methoxy.

2. Please revise the dose of pectine recommended by the manufacturer. Some recommend reconstituting 3.5- 5 gr/d by its consistency and osmotic load to treat constipation.

3. Please explain why in healthy adults inflammatory cytokines were augmented and anti- inflammatory IL-10 diminished.

4. Please revise any literature about appropriate outcomes for clinical trials, establishing those relevant for patients and priorized by core outcome set (COS). Consider the clinical over statistical relevance.

5. Please explain and COS- based, why MRI is optional, not mandatory.

6. Please show the sample size calculation

7. Complete in appropriate manner the tables (revise bullets, spaces, and other details).

8. Since my point of view, I consider that you need a pilot study with dose acceptability by participants.

Thank you

==============================

Please submit your revised manuscript by Jul 03 2026 11:59PM. If you will need more time than this to complete your revisions, please reply to this message or contact the journal office at plosone@plos.org. When you're ready to submit your revision, log on to https://www.editorialmanager.com/pone/ and select the 'Submissions Needing Revision' folder to locate your manuscript file.

Please include the following items when submitting your revised manuscript:

- A letter that responds to each point raised by the academic editor and reviewer(s). You should upload this letter as a separate file labeled 'Response to Reviewers'.
- A marked-up copy of your manuscript that highlights changes made to the original version. You should upload this as a separate file labeled 'Revised Manuscript with Track Changes'.
- An unmarked version of your revised paper without tracked changes. You should upload this as a separate file labeled 'Manuscript'.

If you would like to make changes to your financial disclosure, please include your updated statement in your cover letter. Guidelines for resubmitting your figure files are available below the reviewer comments at the end of this letter.

If applicable, we recommend that you deposit your laboratory protocols in protocols.io to enhance the reproducibility of your results. Protocols.io assigns your protocol its own identifier (DOI) so that it can be cited independently in the future. For instructions see: https://journals.plos.org/plosone/s/submission-guidelines#loc-laboratory-protocols. Additionally, PLOS ONE offers an option for publishing peer-reviewed Lab Protocol articles, which describe protocols hosted on protocols.io. Read more information on sharing protocols at https://plos.org/protocols?utm_medium=editorial-email&utm_source=authorletters&utm_campaign=protocols.

As the corresponding author, your ORCID iD is verified in the submission system and will appear in the published article. PLOS supports the use of ORCID, and we encourage all coauthors to register for an ORCID iD and use it as well. Please encourage your coauthors to verify their ORCID iD within the submission system before final acceptance, as unverified ORCID iDs will not appear in the published article. *Only*  the individual author can complete the verification step; PLOS staff *cannot*  verify ORCID iDs on behalf of authors.

We look forward to receiving your revised manuscript.

Kind regards,

Sophia Eugenia Martínez-Vázquez, Ph.D.

Academic Editor

PLOS One

Journal Requirements:

When submitting your revision, we need you to address these additional requirements.

1. Please ensure that your manuscript meets PLOS ONE's style requirements, including those for file naming. The PLOS ONE style templates can be found at

https://journals.plos.org/plosone/s/file?id=wjVg/PLOSOne_formatting_sample_main_body.pdf and

https://journals.plos.org/plosone/s/file?id=ba62/PLOSOne_formatting_sample_title_authors_affiliations.pdf.

2. Thank you for submitting your clinical trial to PLOS ONE and for providing the name of the registry and the registration number. The information in the registry entry suggests that your trial was registered after patient recruitment began. PLOS ONE strongly encourages authors to register all trials before recruiting the first participant in a study.

As per the journal’s editorial policy, please include in the Methods section of your paper:

a) your reasons for your delay in registering this study (after enrolment of participants started);

b) confirmation that all related trials are registered by stating: “The authors confirm that all ongoing and related trials for this drug/intervention are registered”.

3. Thank you for stating the following financial disclosure:

“This study is funded by the National Institute of Health Research (NIHR) Nottingham Biomedical Research Centre award IS-BRC-1215-20003. “

Please state what role the funders took in the study.  If the funders had no role, please state: "The funders had no role in study design, data collection and analysis, decision to publish, or preparation of the manuscript."

If this statement is not correct you must amend it as needed.

Please include this amended Role of Funder statement in your cover letter; we will change the online submission form on your behalf.

4. Thank you for stating the following in the Competing Interests section:

“GPA has received consulting fees from Amryth, Agios, DNDi, PureTech LYT Inc, Pfizer Inc, GlaxoSmithKline, Clinipace, Merck Healthcare KGaA, JnJ, Suzhou MDCE Co Ltd, SynOx Therapies, Novartis Pharma, AstraZeneca and BenevolentAI Bio, paid to the University of Nottingham and has received investigator-led research funding from Ipsen Ltd and Pfizer Inc. AMV is affiliated with commercial entity(ies) or organisation(s) not related to this particular study but related to a branch of medicine. Specifically, AMV is a member of the Chuckling Goat (a nutrition company) Scientific Advisory Board (SAB). AMV. is also a member of the SABs for OLIPOP Inc. These are not related to the study and had no role in the design of the study; in the collection, analyses, or interpretation of data; in the writing of the manuscript; or in the decision to publish the results. All other authors declare that the research was conducted in the absence of any commercial or financial relationships that could be construed as a potential conflict of interest. “

We note that one or more of the authors are employed by a commercial company:

a.        Please provide an amended Funding Statement declaring this commercial affiliation, as well as a statement regarding the Role of Funders in your study. If the funding organization did not play a role in the study design, data collection and analysis, decision to publish, or preparation of the manuscript and only provided financial support in the form of authors' salaries and/or research materials, please review your statements relating to the author contributions, and ensure you have specifically and accurately indicated the role(s) that these authors had in your study. You can update author roles in the Author Contributions section of the online submission form.

Please also include the following statement within your amended Funding Statement.

“The funder provided support in the form of salaries for authors [insert relevant initials], but did not have any additional role in the study design, data collection and analysis, decision to publish, or preparation of the manuscript. The specific roles of these authors are articulated in the ‘author contributions’ section.”

If your commercial affiliation did play a role in your study, please state and explain this role within your updated Funding Statement.

b. Please also provide an updated Competing Interests Statement declaring this commercial affiliation along with any other relevant declarations relating to employment, consultancy, patents, products in development, or marketed products, etc.

Within your Competing Interests Statement, please confirm that this commercial affiliation does not alter your adherence to all PLOS ONE policies on sharing data and materials by including the following statement: "This does not alter our adherence to  PLOS ONE policies on sharing data and materials.” (as detailed online in our guide for authors http://journals.plos.org/plosone/s/competing-interests) . If this adherence statement is not accurate and  there are restrictions on sharing of data and/or materials, please state these. Please note that we cannot proceed with consideration of your article until this information has been declared.

Please include both an updated Funding Statement and Competing Interests Statement in your cover letter. We will change the online submission form on your behalf.

5. Please be informed that funding information should not appear in the Acknowledgments section or other areas of your manuscript. We will only publish funding information present in the Funding Statement section of the online submission form. Please remove any funding-related text from the manuscript.

6.Your ethics statement should only appear in the Methods section of your manuscript. If your ethics statement is written in any section besides the Methods, please move it to the Methods section and delete it from any other section. Please ensure that your ethics statement is included in your manuscript, as the ethics statement entered into the online submission form will not be published alongside your manuscript.

7.We note that the original protocol file you uploaded contains a confidentiality notice indicating that the protocol may not be shared publicly or be published. Please note, however, that the PLOS Editorial Policy requires that the original protocol be published alongside your manuscript in the event of acceptance. Please note that should your paper be accepted, all content including the protocol will be published under the Creative Commons Attribution (CC BY) 4.0 license, which means that it will be freely available online, and any third party is permitted to access, download, copy, distribute, and use these materials in any way, even commercially, with proper attribution.

Therefore, we ask that you please seek permission from the study sponsor or body imposing the restriction on sharing this document to publish this protocol under CC BY 4.0 if your work is accepted. We kindly ask that you upload a formal statement signed by an institutional representative clarifying whether you will be able to comply with this policy. Additionally, please upload a clean copy of the protocol with the confidentiality notice (and any copyrighted institutional logos or signatures) removed.

8.We note that the original protocol that you have uploaded as a Supporting Information file contains an institutional logo. As this logo is likely copyrighted, we ask that you please remove it from this file and upload an updated version upon resubmission

9. If the reviewer comments include a recommendation to cite specific previously published works, please review and evaluate these publications to determine whether they are relevant and should be cited. There is no requirement to cite these works unless the editor has indicated otherwise.

[Note: HTML markup is below. Please do not edit.]

Reviewers' comments:

Reviewer's Responses to Questions

**Comments to the Author**

1. Does the manuscript provide a valid rationale for the proposed study, with clearly identified and justified research questions?

The research question outlined is expected to address a valid academic problem or topic and contribute to the base of knowledge in the field.

Reviewer #1: Yes

Reviewer #2: Partly

**********

2. Is the protocol technically sound and planned in a manner that will lead to a meaningful outcome and allow testing the stated hypotheses?

The manuscript should describe the methods in sufficient detail to prevent undisclosed flexibility in the experimental procedure or analysis pipeline, including sufficient outcome-neutral conditions (e.g. necessary controls, absence of floor or ceiling effects) to test the proposed hypotheses and a statistical power analysis where applicable. As there may be aspects of the methodology and analysis which can only be refined once the work is undertaken, authors should outline potential assumptions and explicitly describe what aspects of the proposed analyses, if any, are exploratory.

Reviewer #1: Partly

Reviewer #2: Partly

**********

3. Is the methodology feasible and described in sufficient detail to allow the work to be replicable?

Descriptions of methods and materials in the protocol should be reported in sufficient detail for another researcher to reproduce all experiments and analyses. The protocol should describe the appropriate controls, sample size calculations, and replication needed to ensure that the data are robust and reproducible.

Reviewer #1: Yes

Reviewer #2: Yes

**********

4. Have the authors described where all data underlying the findings will be made available when the study is complete?

The PLOS Data policy requires authors to make all data underlying the findings described in their manuscript fully available without restriction, with rare exception, at the time of publication. The data should be provided as part of the manuscript or its supporting information, or deposited to a public repository. For example, in addition to summary statistics, the data points behind means, medians and variance measures should be available. If there are restrictions on publicly sharing data—e.g. participant privacy or use of data from a third party—those must be specified.

Reviewer #1: Yes

Reviewer #2: Yes

**********

5. Is the manuscript presented in an intelligible fashion and written in standard English?

PLOS ONE does not copyedit accepted manuscripts, so the language in submitted articles must be clear, correct, and unambiguous. Any typographical or grammatical errors should be corrected at revision, so please note any specific errors here.

Reviewer #1: Yes

Reviewer #2: Yes

**********

6. Review Comments to the Author

Please use the space provided to explain your answers to the questions above and, if applicable, provide comments about issues authors must address before this protocol can be accepted for publication. You may also include additional comments for the author, including concerns about research or publication ethics.

You may also provide optional suggestions and comments to authors that they might find helpful in planning their study.

(Please upload your review as an attachment if it exceeds 20,000 characters)

Reviewer #1: The elements of this study protocol such as background, rationale , eligibility criteria etc. are provided and will require review of course. My focus is on the statistical aspects of the study. This has a good Statistical Design and Analysis plan and is written as such. The data management and quality control (data validation) looks well in place. The protocol provided in the supplement is well written and helpful. However, there are clarifications and detail which need attention in the sample size and analysis sections as follows:

1. The sample size calculation needs to be checked. It appears that the prior SD assumption seems to be the same for both groups at about 0.036 to arrive at the sample size given by the investigators. This should be an unequal variance calculation according to the background data provided. This will not change the sample size much as such, but should be checked.

2. The missing data assumption being handled by the mixed model approach is reasonable. However, the MAR assumption should have a bit more justification. With regard to recruitment and retention a retention strategy for this type of study should be in place even though the follow up time is short and there is anticipated frequent subject contact. It may minimize the missing data .

3. The analysis via mixed models with random effects looks reasonable. However, the use of a multivariate non parametric alternative should be detailed in the case of non normality.

4. Looking at Figure 1, there are many assessments. Even in this exploratory framework with a small sample size, many analysis ( 6 markers plus the secondary effects) may call for a multiple comparison adjustment of the p-values which the investigators should consider.

Reviewer #2: General comments

The objective of this study is to investigate the effects of daily LM pectin ingestion on inflammatory pathways that drive disease progression, and also to assess changes in anthropometric measures; to evaluate changes in general metabolic indicators, including fasting blood glucose and blood-based biomarkers relevant to MASLD activity and fibrosis. Given the recent growing interest in the relationship between the intake of fermentable dietary fiber and the prevention of MASLD, this study protocol may be of significant importance. However, I have major concerns about this manuscript, particularly regarding the rationale for the dietary fiber intake levels and the lack of data on dietary fiber intake from daily meals.

1. In addition to the dietary fiber intake from regular meals, a daily dose of 15 g of LM pectin may be excessive. Please provide the basis for determining this dosage. The issue of diarrhea must also be taken into account.

2. The placebo formulation must also contain the same amount of dietary fiber, such as cellulose. There is a concern that the effects of adding LM pectin may be confounded by the effects of simply increasing the amount of dietary fiber.

3. It is essential to examine dietary fiber intake, particularly soluble dietary fiber intake, based on daily food records kept throughout the study period.

Specific comments

1. l.83 The author states that HM pectin is generally difficult to ferment in the human body. However, there are reports indicating that HM pectin is a type of fermentable dietary fiber. Please provide evidence that HM pectin has low fermentability.

**********

7. PLOS authors have the option to publish the peer review history of their article (what does this mean?). If published, this will include your full peer review and any attached files.

If you choose “no”, your identity will remain anonymous but your review may still be made public.

**Do you want your identity to be public for this peer review?**  For information about this choice, including consent withdrawal, please see our Privacy Policy.

Reviewer #1: No

Reviewer #2: **Yes:**  Seiichiro Aoe

**********

[NOTE: If reviewer comments were submitted as an attachment file, they will be attached to this email and accessible via the submission site. Please log into your account, locate the manuscript record, and check for the action link "View Attachments". If this link does not appear, there are no attachment files.]

To ensure your figures meet our technical requirements, please review our figure guidelines: https://journals.plos.org/plosone/s/figures

You may also use PLOS’s free figure tool, NAAS, to help you prepare publication quality figures: https://journals.plos.org/plosone/s/figures#loc-tools-for-figure-preparation.

NAAS will assess whether your figures meet our technical requirements by comparing each figure against our figure specifications.

# Author comment: Author response to Decision Letter 1

**DOI:** 10.1371/journal.pone.0352397.r002

2 Jun 2026

Responses to the Academic Editor

Comment 1

Please revise the term methoxyl instead of methoxy.

Response:

We thank the Editor for this observation. The terminology has been revised throughout the manuscript from “methoxy” to “methoxyl” where appropriate to align with accepted pectin chemistry nomenclature.

Changes were made throughout the manuscript.

Comment 2

Please revise the dose of pectin recommended by the manufacturer. Some recommend reconstituting 3.5–5 g/day by its consistency and osmotic load to treat constipation.

Response:

We appreciate this important comment. The selected dose of 15 g/day was based on previously published human supplementation studies demonstrating acceptable tolerability and measurable biological effects at doses ranging from 15–20 g/day of low-methoxyl pectin. Furthermore, the intervention dose was developed in consultation with dietetic specialists during protocol development and was considered appropriate within the context of habitual fibre intakes that are typically below recommended levels in the general population. Current nutritional guidance recommends a daily dietary fibre intake of approximately 25–30 g/day for adults. In addition, the intervention is intended to investigate mechanistic metabolic and inflammatory effects rather than constipation treatment. Participants will be monitored throughout the study for gastrointestinal symptoms, and exclusion criteria were designed to minimise risks among individuals with pre-existing gastrointestinal disorders. Additional justification for dose selection and safety monitoring has now been added to the manuscript.

Revisions added to the Introduction and Safety Considerations sections.

Comment 3

Please explain why in healthy adults, inflammatory cytokines were augmented and anti-inflammatory IL-10 diminished.

Response:

We thank the Editor for highlighting the need for clarification. The cited pilot study reported reductions in pro-inflammatory cytokines together with an increase in the anti-inflammatory cytokine IL-10 following LM pectin supplementation. The manuscript has now been revised to improve clarity and to briefly describe the proposed mechanistic pathways underlying these immunomodulatory effects, including microbiota-mediated short-chain fatty acid production and improved gut barrier function.

The relevant section in the Introduction has been revised for clarity and accuracy.

Comment 4

Please revise any literature about appropriate outcomes for clinical trials, establishing those relevant for patients and prioritized by core outcome set (COS). Consider the clinical over statistical relevance.

Response:

We appreciate this valuable suggestion. Additional rationale has now been included to justify the selected outcomes based on mechanistic relevance to MASLD progression, inflammation, metabolic dysfunction, and patient-centred non-invasive disease assessment. We also clarified that the study is exploratory and hypothesis-generating, aiming to inform future larger clinical trials and core outcome development in MASLD nutritional interventions.

Additional text has been incorporated into the Outcomes and Discussion sections.

Comment 5

Please explain and COS-based, why MRI is optional, not mandatory.

Response:

We thank the Editor for this important point. MRI assessments were included as an optional sub-study due to logistical considerations, participant burden, scanner availability, and MRI contraindications that may limit participation. The primary and secondary outcomes of the trial rely on biochemical, anthropometric, FibroScan™, and clinical measures collected from all participants. MRI-derived measures are exploratory mechanistic outcomes intended to provide additional insight into hepatic fat distribution and intestinal permeability.

Clarification has been added to the Methods section.

Comment 6

Please show the sample size calculation.

Response:

We thank the Editor for this comment. The sample size calculation has now been expanded and clarified in the Statistical Analysis subsection, including assumptions regarding effect size, variability estimates, significance level, statistical power, and anticipated dropout rate.

The Statistical Analysis section has been revised accordingly.

Comment 7

Complete in appropriate manner the tables (revise bullets, spaces, and other details).

Response:

We appreciate this observation. All tables and formatting details have been carefully revised for consistency with PLOS ONE formatting requirements.

Formatting corrections were made throughout the manuscript.

Comment 8

Since my point of view, I consider that you need a pilot study with dose acceptability by participants.

Response:

We appreciate this suggestion. The intervention dose and study procedures were informed by previously published human studies evaluating LM pectin supplementation as well as prior dietary intervention studies conducted by the research team using similar methodologies. Based on these existing data and the low-risk nature of the intervention, an additional pilot acceptability study was not considered essential before conducting the present mechanistic trial.

Responses to Reviewer #1

Comment 1

The sample size calculation needs to be checked. It appears that the prior SD assumption seems to be the same for both groups.

Response:

We thank the reviewer for this important observation. The sample size calculation has been revisited using unequal variance assumptions based on the referenced pilot data. The MRI sub-study sample size calculation was based on previously published quantitative MRI research evaluating changes in small bowel permeability following indomethacin provocation in healthy volunteers, which reported different standard deviations between groups (0.115 ± 0.063 s vs 0.070 ± 0.036 s). Therefore, unequal variance assumptions were used in the calculation. Recalculation did not materially alter the planned recruitment target. The sample size justification has now been clarified in the revised manuscript.

Revisions added to the Statistical Analysis section.

Comment 2

The MAR assumption should have more justification. A retention strategy should also be included.

Response:

We appreciate this helpful suggestion. Additional justification regarding the missing-at-random assumption has now been included. Furthermore, we added details regarding participant retention strategies, including regular participant contact, flexible scheduling, reminder communications, and reimbursement arrangements intended to minimise missing data and improve adherence.

Additional text added to the Statistical Analysis and Study Procedures sections.

Comment 3

The use of a multivariate non-parametric alternative should be detailed in the case of non-normality.

Response:

We thank the reviewer for this recommendation. The statistical analysis section has been revised to clarify that, where substantial deviation from normality persists despite transformation, appropriate non-parametric longitudinal or rank-based sensitivity analyses will be considered.

Clarification added to the Statistical Analysis section.

Comment 4

Multiple comparison adjustment should be considered.

Response:

We appreciate this important point. The manuscript has now been revised to clarify that secondary and exploratory analyses will be interpreted cautiously, and appropriate multiplicity adjustment approaches, including false discovery rate correction where appropriate, will be considered.

Additional text added to the Statistical Analysis section.

Responses to Reviewer #2

General Comment

The rationale for dietary fibre intake levels and lack of dietary fibre intake data from daily meals are major concerns.

Response:

We thank the reviewer for these important observations. We agree that habitual dietary fibre intake may influence study outcomes. At baseline, participants will complete study case report forms (CRFs) that include questions regarding current probiotic use and habitual dietary fibre intake. This information will be used to characterise participants’ baseline dietary patterns and identify major changes during the intervention period. In addition, population dietary intake data indicate that average fibre consumption in the general population is commonly below the recommended intake range of 25–30 g/day. The selected intervention dose was therefore designed to remain within a physiologically achievable dietary range while enabling assessment of mechanistic effects. The intervention dose was also developed in consultation with dietetic specialists during study design.

Additional details have been added to the Outcome Assessments and Study Procedures sections.

Comment 1

A daily dose of 15 g of LM pectin may be excessive. Please provide the basis for determining this dosage. The issue of diarrhea must also be taken into account.

Response:

We appreciate this important comment. The selected dose was informed by previous human supplementation studies demonstrating acceptable tolerability and measurable biological effects at doses ranging from 15–20 g/day of LM pectin. Participants with gastrointestinal disorders or sensitivities are excluded, and gastrointestinal symptoms will be monitored throughout the intervention. Safety considerations regarding bloating, gastrointestinal discomfort, and diarrhoea have now been further clarified.

Additional rationale added to the Introduction and Safety Considerations sections.

Comment 2

The placebo formulation must also contain the same amount of dietary fibre.

Response:

We thank the reviewer for this thoughtful suggestion. The placebo was intentionally designed without fermentable fibre to isolate the mechanistic effects of LM pectin supplementation on inflammatory and microbiome-related pathways. Inclusion of alternative fibre sources, including cellulose, may independently influence gastrointestinal physiology, microbial composition, and metabolic outcomes, thereby introducing additional confounding effects. The placebo was therefore selected to match flavour, appearance, and mode of administration while minimising biological activity.

This rationale has now been clarified in the Intervention and Comparator section.

Comment 3

It is essential to examine dietary fibre intake from daily food records.

Response:

We fully agree with the reviewer. At baseline, participants will complete study case report forms (CRFs) that include questions regarding current probiotic use and habitual dietary fibre intake. This information will be used to characterise participants’ baseline dietary patterns and identify major changes during the intervention period.

Additional methodological details have been added to the manuscript.

Specific Comment

Please provide evidence that HM pectin has low fermentability.

Response:

We thank the reviewer for this clarification. The manuscript wording has been revised to avoid overstatement. We now state that HM pectin may demonstrate lower fermentability relative to LM pectin depending on degree of esterification and microbial accessibility, supported by the relevant literature.

The Introduction section has been revised accordingly.

Attachment Submitted filename: Respond to editors and reviewers.docx

# Peer review: Decision Letter 1

**DOI:** 10.1371/journal.pone.0352397.r003

**Authors:** Sophia Martínez-Vázquez, Sophia Martínez-Vázquez

9 Jun 2026

The impact of pectin supplementation on systemic inflammation pathways, gut microbiome, and metabolic health in patients with Metabolic Dysfunction-Associated Steatotic Liver Disease (MASLD): A study protocol for a randomised controlled trial

PONE-D-26-17120R1

Dear Dr. Noor Kifah Al-Tameemi,

We’re pleased to inform you that your manuscript has been judged scientifically suitable for publication and will be formally accepted for publication once it meets all outstanding technical requirements.

Within one week, you’ll receive an e-mail detailing the required amendments. When these have been addressed, you’ll receive a formal acceptance letter and your manuscript will be scheduled for publication.

An invoice will be generated when your article is formally accepted. Please note, if your institution has a publishing partnership with PLOS and your article meets the relevant criteria, all or part of your publication costs will be covered. Please make sure your user information is up-to-date by logging into Editorial Manager at Editorial Manager® and clicking the ‘Update My Information' link at the top of the page. For questions related to billing, please contact billing support.

If your institution or institutions have a press office, please notify them about your upcoming paper to help maximize its impact. If they’ll be preparing press materials, please inform our press team as soon as possible -- no later than 48 hours after receiving the formal acceptance. Your manuscript will remain under strict press embargo until 2 pm Eastern Time on the date of publication. For more information, please contact onepress@plos.org.

Kind regards,

Sophia Eugenia Martínez-Vázquez, Ph.D.

Academic Editor

PLOS One

Additional Editor Comments (optional):

Dear Authors,

Thank you for your responses, they are adequately. Congratulations.

Reviewers' comments:

Reviewer's Responses to Questions

**Comments to the Author**

1. Does the manuscript provide a valid rationale for the proposed study, with clearly identified and justified research questions?

The research question outlined is expected to address a valid academic problem or topic and contribute to the base of knowledge in the field.

Reviewer #1: Yes

**********

2. Is the protocol technically sound and planned in a manner that will lead to a meaningful outcome and allow testing the stated hypotheses?

The manuscript should describe the methods in sufficient detail to prevent undisclosed flexibility in the experimental procedure or analysis pipeline, including sufficient outcome-neutral conditions (e.g. necessary controls, absence of floor or ceiling effects) to test the proposed hypotheses and a statistical power analysis where applicable. As there may be aspects of the methodology and analysis which can only be refined once the work is undertaken, authors should outline potential assumptions and explicitly describe what aspects of the proposed analyses, if any, are exploratory.

Reviewer #1: Yes

**********

3. Is the methodology feasible and described in sufficient detail to allow the work to be replicable?

Descriptions of methods and materials in the protocol should be reported in sufficient detail for another researcher to reproduce all experiments and analyses. The protocol should describe the appropriate controls, sample size calculations, and replication needed to ensure that the data are robust and reproducible.

Reviewer #1: Yes

**********

4. Have the authors described where all data underlying the findings will be made available when the study is complete?

The PLOS Data policy requires authors to make all data underlying the findings described in their manuscript fully available without restriction, with rare exception, at the time of publication. The data should be provided as part of the manuscript or its supporting information, or deposited to a public repository. For example, in addition to summary statistics, the data points behind means, medians and variance measures should be available. If there are restrictions on publicly sharing data—e.g. participant privacy or use of data from a third party—those must be specified.

Reviewer #1: Yes

**********

5. Is the manuscript presented in an intelligible fashion and written in standard English?

PLOS ONE does not copyedit accepted manuscripts, so the language in submitted articles must be clear, correct, and unambiguous. Any typographical or grammatical errors should be corrected at revision, so please note any specific errors here.

Reviewer #1: Yes

**********

6. Review Comments to the Author

Please use the space provided to explain your answers to the questions above and, if applicable, provide comments about issues authors must address before this protocol can be accepted for publication. You may also include additional comments for the author, including concerns about research or publication ethics.

You may also provide optional suggestions and comments to authors that they might find helpful in planning their study.

(Please upload your review as an attachment if it exceeds 20,000 characters)

Reviewer #1: All comments have been addressed and the revisions noted in the manuscript.

XXXXXXXXXXXXXXXXXXXXXXXXXXXXXXXXXXXXXXXXXXXXXXXXXXXXXXXXXXXXXXXXXXXXXXXXXXXXXXXXXXXXXXXXXXXXXXXXXXXXXXXXXXXXXXXXXXXXXXXXXXXXXXXXX

**********

7. PLOS authors have the option to publish the peer review history of their article (what does this mean?). If published, this will include your full peer review and any attached files.

If you choose “no”, your identity will remain anonymous but your review may still be made public.

**Do you want your identity to be public for this peer review?**  For information about this choice, including consent withdrawal, please see our Privacy Policy.

Reviewer #1: No

**********

# Editor report: Acceptance letter

**DOI:** 10.1371/journal.pone.0352397.r004

**Authors:** Sophia Martínez-Vázquez, Sophia Martínez-Vázquez

PONE-D-26-17120R1

PLOS One

Dear Dr. Al-Tameemi,

I'm pleased to inform you that your manuscript has been deemed suitable for publication in PLOS One. Congratulations! Your manuscript is now being handed over to our production team.

At this stage, our production department will prepare your paper for publication. This includes ensuring the following:

* All references, tables, and figures are properly cited

* All relevant supporting information is included in the manuscript submission,

* There are no issues that prevent the paper from being properly typeset

You will receive further instructions from the production team, including instructions on how to review your proof when it is ready. Please keep in mind that we are working through a large volume of accepted articles, so please give us a few days to review your paper and let you know the next and final steps.

Lastly, if your institution or institutions have a press office, please let them know about your upcoming paper now to help maximize its impact. If they'll be preparing press materials, please inform our press team within the next 48 hours. Your manuscript will remain under strict press embargo until 2 pm Eastern Time on the date of publication. For more information, please contact onepress@plos.org.

You will receive an invoice from PLOS for your publication fee after your manuscript has reached the completed accept phase. If you receive an email requesting payment before acceptance or for any other service, this may be a phishing scheme. Learn how to identify phishing emails and protect your accounts at https://explore.plos.org/phishing.

If we can help with anything else, please email us at customercare@plos.org.

Thank you for submitting your work to PLOS ONE and supporting open access.

Kind regards,

PLOS ONE Editorial Office Staff

on behalf of

Dr. Sophia Eugenia Martínez-Vázquez

Academic Editor

PLOS One
